# Gating Topology of the Proton-Coupled Oligopeptide Symporters

**DOI:** 10.1016/j.str.2014.12.012

**Published:** 2015-02-03

**Authors:** Philip W. Fowler, Marcella Orwick-Rydmark, Sebastian Radestock, Nicolae Solcan, Patricia M. Dijkman, Joseph A. Lyons, Jane Kwok, Martin Caffrey, Anthony Watts, Lucy R. Forrest, Simon Newstead

**Affiliations:** 1 Department of Biochemistry, University of Oxford, South Parks Road, Oxford OX1 3QU, UK; 2 Max Planck Institute of Biophysics, Max-von-Laue-Straße 3, Frankfurt am Main, Germany; 3 School of Biochemistry and Immunology, Trinity College Dublin, Dublin 2, Ireland

## Abstract

Proton-coupled oligopeptide transporters belong to the major facilitator superfamily (MFS) of membrane transporters. Recent crystal structures suggest the MFS fold facilitates transport through rearrangement of their two six-helix bundles around a central ligand binding site; how this is achieved, however, is poorly understood. Using modeling, molecular dynamics, crystallography, functional assays, and site-directed spin labeling combined with double electron-electron resonance (DEER) spectroscopy, we present a detailed study of the transport dynamics of two bacterial oligopeptide transporters, PepT_So_ and PepT_St_. Our results identify several salt bridges that stabilize outward-facing conformations and we show that, for all the current structures of MFS transporters, the first two helices of each of the four inverted-topology repeat units form half of either the periplasmic or cytoplasmic gate and that these function cooperatively in a scissor-like motion to control access to the peptide binding site during transport.

## Highlights

• New higher-resolution structure of PepT_So_
• Salt bridges stabilizing outward-facing conformations are identified
• The conserved prolines in helix 8 are shown to be important
• The first two helices in each inverted-topology repeat form part of a gate

## Graphical Abstract

**Figure:**
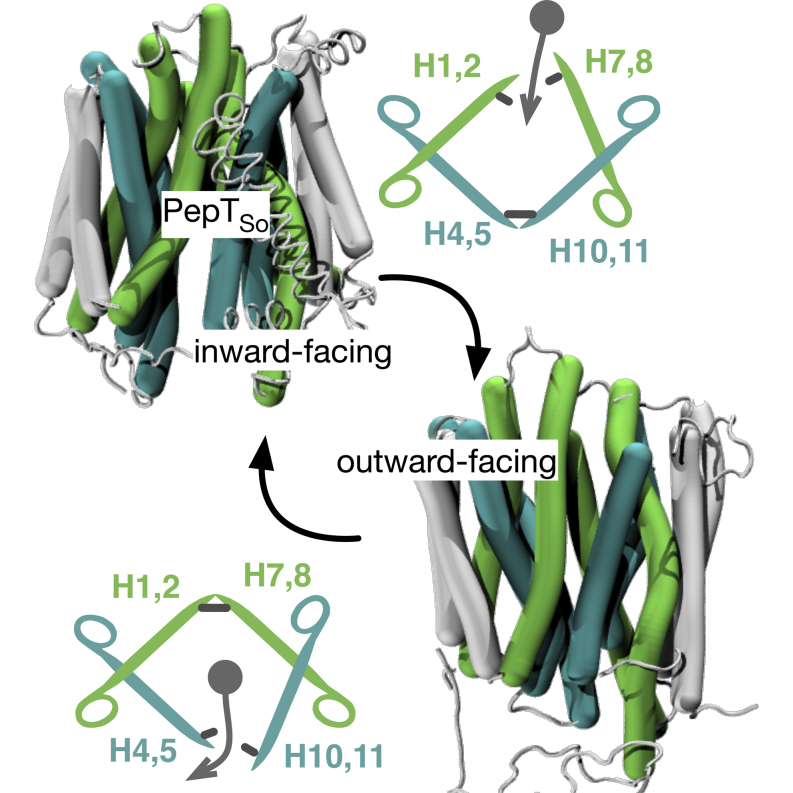

## Teaser

Fowler et al. use biophysical and modeling approaches to identify salt bridges in two peptide transporters that stabilize their outward-facing conformations. Their results also suggest that the first two helices in each inverted-topology repeat unit forms part of either of the two gates.

## Introduction

Peptide transport is the main route through which the body absorbs and retains dietary protein and hence plays an important role in human physiology (Steinhardt and Adibi, 1986). The combined action of acid hydrolysis in the stomach and nonspecific peptidases in the small intestine breaks down ingested protein into peptide fragments and free amino acids. The resulting di- and tripeptides are then actively transported across the intestinal brush border membrane by the integral membrane peptide transporter, PepT1 (Fei et al., 1994; Leibach and Ganapathy, 1996). PepT1 recognizes a diverse range of small peptides and is also responsible for the absorption of many orally administered drugs, including β-lactam antibiotics and a growing number of peptiditic prodrugs (Luckner and Brandsch, 2005; Pieri et al., 2009; Brandsch, 2009). We do not yet fully understand the mechanism by which PepT1 recognizes and transports molecules into the cell, and this lack of knowledge is hampering the modification of drugs to improve their pharmacokinetic profiles.

PepT1 is a member of the POT family of proton-dependent oligopeptide transporters (TC 2.A.17), which itself belongs to the much larger major facilitator superfamily (MFS) of secondary active transport proteins (Reddy et al., 2012). POT family transporters contain either 12 or 14 transmembrane α helices. Structures of four bacterial members of the POT family have been determined: these are PepT_So_ (Newstead et al., 2011), from the bacterium *Shewanella oniedensis*; PepT_St_ (Solcan et al., 2012; Lyons et al., 2014), from the thermophilic mesophile *Streptococcus thermophilus*; and, more recently, GkPOT from the bacterium *Geobacillus kaustophilus* (Doki et al., 2013); PepT_So2_, also from *Shewanella oniedensis* (Guettou et al., 2013). Biochemical studies on the bacterial POT family have revealed that these proteins operate in a similar way to their mammalian counterparts, with many of the functionally important residues conserved (Figure S8) (Daniel et al., 2006; Harder et al., 2008).

The MFS is the largest superfamily of secondary active transporters, containing over 70 different families (Reddy et al., 2012). Structures of members belonging to several MFS families reveal a common fold consisting of two bundles of six transmembrane (TM) α helices that come together to form a “⋀”- or “V”-shaped transporter with a central substrate binding site (Figure S1) (Hirai et al., 2002; Abramson et al., 2003; Huang et al., 2003; Yin et al., 2006; Dang et al., 2010; Newstead et al., 2011; Solcan et al., 2012; Sun et al., 2012; Yan et al., 2013; Pedersen et al., 2013; Quistgaard et al., 2013; Guettou et al., 2013; Doki et al., 2013; Jiang et al., 2013; Deng et al., 2014). Not only are the two six-helix bundles structurally similar but there are also conserved sequence motifs between helices 2 and 3 in the N-terminal domain and between helices 7 and 8 in the C-terminal domain (Pao et al., 1998; Saier et al., 1999).

**Figure 1:**
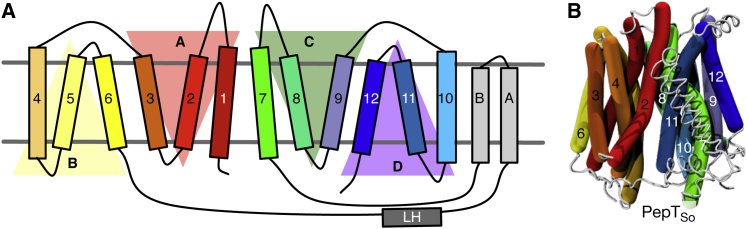
Proton Oligopeptide Symporters Comprise Four Inverted-Topology Repeat Units (A) Each inverted-topology repeat unit (labeled A–D) is made up of three transmembrane α helices (labeled 1–12) (Radestock and Forrest, 2011). (B) The inward-occluded structure of PepT_So_ rendered using curved cylinders (Dahl et al., 2012) to illustrate their intrinsic kinks and bends and colored according to the same scheme as in (A).

Recently it was proposed that each bundle can be further divided into two inverted-topology repeat units (four in total, labeled A–D, Figure 1) (Hvorup and Saier, 2002; Radestock and Forrest, 2011), revealing a more fundamental level of symmetry. The first three helices of the protein form repeat unit A; its structure is related to the second three helices (repeat unit B) via a two-fold symmetry axis running through the center of the six-helical bundle and parallel to the plane of the membrane. A similar pair of repeat units is found within the second six-helical bundle (repeat units C and D). These proteins can also be considered as comprising two six-helix repeats with inverted topologies. That is, units A and D comprise a noncontiguous six-helix repeat that has opposite transmembrane topology from the contiguous units B and C. There is a significant difference in the angle of the C-terminal three-helix units relative to the N-terminal three-helix units in the inward-open structure of LacY, such that the two six-helix inverted repeats are asymmetric (in that state). This feature means that the inward-open conformation of LacY can be converted to an outward-open conformation by swapping the structures of each pair of repeat units (Radestock and Forrest, 2011), suggesting that an “asymmetry-exchange” mechanism underlies the overall conformational change. This method has been shown to reproduce key features of alternate conformations for several other transporter architectures (Schushan et al., 2012; Crisman et al., 2009; Liao et al., 2012; Radestock and Forrest, 2011).

Peptide transporters are proton-coupled symporters and use the inwardly directed proton electrochemical gradient to drive peptide uptake into the cell. The alternating-access mechanism (Jardetzky, 1966) predicts that they (1) facilitate transport by moving between outward- and inward-open conformations, (2) can only exchange between these conformations when in either the apo state or when both a substrate and one or more protons are bound, and (3) cannot form a continuous pore across the bilayer. Peptide transporters must therefore prevent access to the binding site(s) from at least one side of the membrane at all times. The rocker-switch mechanism of MFS transport extended the alternating-access mechanism by proposing that the bound ligand becomes alternately exposed to either side of the membrane by the rocking of the two symmetry-related six-helical bundles around the central binding site (Huang et al., 2003; Law et al., 2008).

To occlude the binding site to either side of the membrane, MFS transporters form gates; these are transient constrictions formed by the close packing of several transmembrane helices. These gates block entry or exit to the central cavity and are stabilized by interactions between transmembrane helices, specifically through key salt bridges, which in turn are controlled by substrate binding and release. Two transmembrane α helices (H1 & H7), one from each six-helical bundle, were identified as forming the periplasmic gate of GlpT (Huang et al., 2003). The structures of PepT_So_ and PepT_St_ further suggested that the periplasmic gate is formed by helices H1 & H2 packing against H7 & H8 (Newstead et al., 2011; Solcan et al., 2012). The structure of PepT_So_ was captured in an asymmetric inward-occluded conformation and its proposed cytoplasmic gate is sufficiently narrow that, although the binding site is contiguous with the intracellular medium, substrates are likely to be sterically impeded (Newstead et al., 2011). By contrast, the structure of PepT_St_ is more symmetric and, as a result, substrates are not prevented from exiting by the cytoplasmic gate. Comparing the structures of PepT_So_ and PepT_St_ in more detail revealed that helices H7, H10, and H11 in the C-terminal bundle of PepT_So_ had shifted relative to the inward-open conformation of PepT_St_. This asymmetry in the motion of the transmembrane α helices is incompatible with a rigid-body rocker-switch model of transport (Newstead et al., 2011; Lyons et al., 2014). Instead it was suggested that a dynamic movement of helices within the two six-helical bundles may be required for the central cavity to be alternately exposed to both sides of the membrane.

Here we present a mechanism for alternating access within the POT family. By systematic analysis of available MFS structures, we show that the cytoplasmic gate is formed by helices H4, H5, H10, and H11, as predicted previously (Newstead et al., 2011; Solcan et al., 2012) and also that the periplasmic gate is formed from the equivalent helices by symmetry, H1, H2, H7, and H8. The first two helices in each of the four repeat units therefore participate in either the periplasmic or the cytoplasmic gate while the third helix is less dynamic. Furthermore, we show that within the POT family, the inward-open and outward-open conformations are stabilized by salt bridges and that kinks introduced into the transmembrane helices by conserved prolines are important in transport. We propose that transport in the POT family can be described by an asymmetric scissor-type motion of the helices in the repeat units, thereby linking the structural symmetries and the alternating-access mechanism in this family of MFS transporters. This provides a more complete picture of the gating topology of the MFS superfamily.

## Results

To investigate the gating of the proton-coupled oligopeptide symporters, we needed representative structures of members of this family in both inward- and outward-facing conformations. The experimental structures of PepT_So_, PepT_St_, PepT_So2_, and GkPOT, however, are all inward facing. We therefore pursued two independent approaches to generating plausible models of both PepT_So_ and PepT_St_ in outward-facing conformations. The first approach was to build outward-facing models of both proteins using the repeat-swapping method (Radestock and Forrest, 2011; Forrest, 2013), while the second was to run molecular dynamics simulations of both proteins with a view to generating outward-facing conformations.

### Outward-Open Conformations of PepT_So_ and PepT_St_

**Figure 2:**
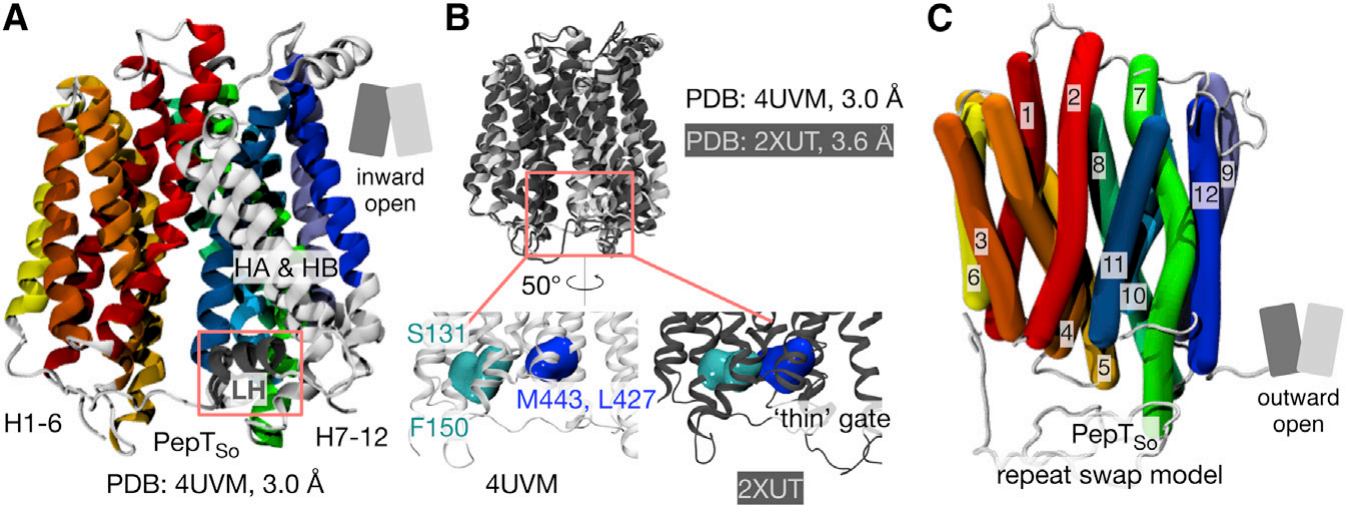
An Inward-Open Structure of the Bacterial Oligopeptide Transporter PepT_So_ (A) The structure of PepT_So_ in an inward-open conformation solved to 3.0 Å using X-ray crystallography. The transmembrane helices are colored from red (H1) to blue (H12) as in Figure 1. The two additional helices found in the bacterial proton oligopeptide transporters, HA and HB, are colored light gray. A lateral helix (LH) found between H6 and HA and not seen in the previous structure is highlighted. The data collection and refinement statistics can be found in Table 1. (B) This new structure of PepT_So_ is broadly similar to that of the lower-resolution inward-occluded structure of PepT_So_ (PDB: 2XUT) (Newstead et al., 2011). The C_α_ RMSD, excluding the HA and HB motif, between both structures is 1.7 Å (394 residues). Some differences can, however, be seen. One of these is the positions of the residues that make up the thin gate; in the new structure these are such that the peptide binding site is accessible to the cytoplasm and hence this structure is inward-open. Additional detail can be found in Figure S2. (C) An outward-open model of PepT_So_, built using the repeat-swapping method. An image of the outward-open model of PepT_St_ is shown in Figure S2.

The repeat-swapping method threads the sequence of repeat unit A onto the structure of repeat unit B, and vice versa, while simultaneously carrying out the same process for the C-terminal half of the protein (units C and D) (Figure 1; Figures S10 and S11), thereby creating a model in the opposing conformation, in this case outward open. The existing inward-occluded structure of PepT_So_ (Newstead et al., 2011) was found to be not suitable for constructing a repeat-swapped model due to asymmetries between the N- and C-terminal halves of the protein, which led to steric hindrance problems during model building. A new crystal structure of PepT_So_ (Protein Data Bank [PDB] ID 4UVM) at a resolution of 3.0 Å was obtained that is both more symmetric and at a higher resolution than the original structure (Figure 2A). Aligning this structure with the original PepT_So_ structure (PDB ID 2XUT) shows that although they are similar, i.e. inward facing, there are several key differences. First, a lateral helix between H6 and HA is resolved for the first time. A similar helix has been identified in the nitrate transporter NRT1.1 (Parker and Newstead, 2014; Sun et al., 2014). Second, the residues that make up the cytoplasmic thin gate adopt different positions (Figure 2B; Figure S2), such that this structure is inward open (the new PepT_So_ structure thus resembles the previously reported inward-open PepT_St_ structure). Third, some weak difference density (*F*_o_ − *F*_c_) is present in a position equivalent to that observed in the previous structure, indicating that this crystal structure may not represent a fully inward-open, ligand-free state. This may explain why the structural differences are smaller than those observed for PepT_St_ (Lyons et al., 2014). Fourth, a solvent-accessible cavity is observed in the new structure that permits protons to access the ExxERF motif on H1 (Figure S2). This motif has previously been shown to play an important role in proton-coupled transport (Solcan et al., 2012). The repeat-swapping method was applied to both this structure of PepT_So_ and the existing inward-open structure of PepT_St_, generating outward-facing models of both proteins (Figure 2C; Figure S2).

### Validating the Outward-Open PepT_So_ Model by DEER Spectroscopy

**Figure 3:**
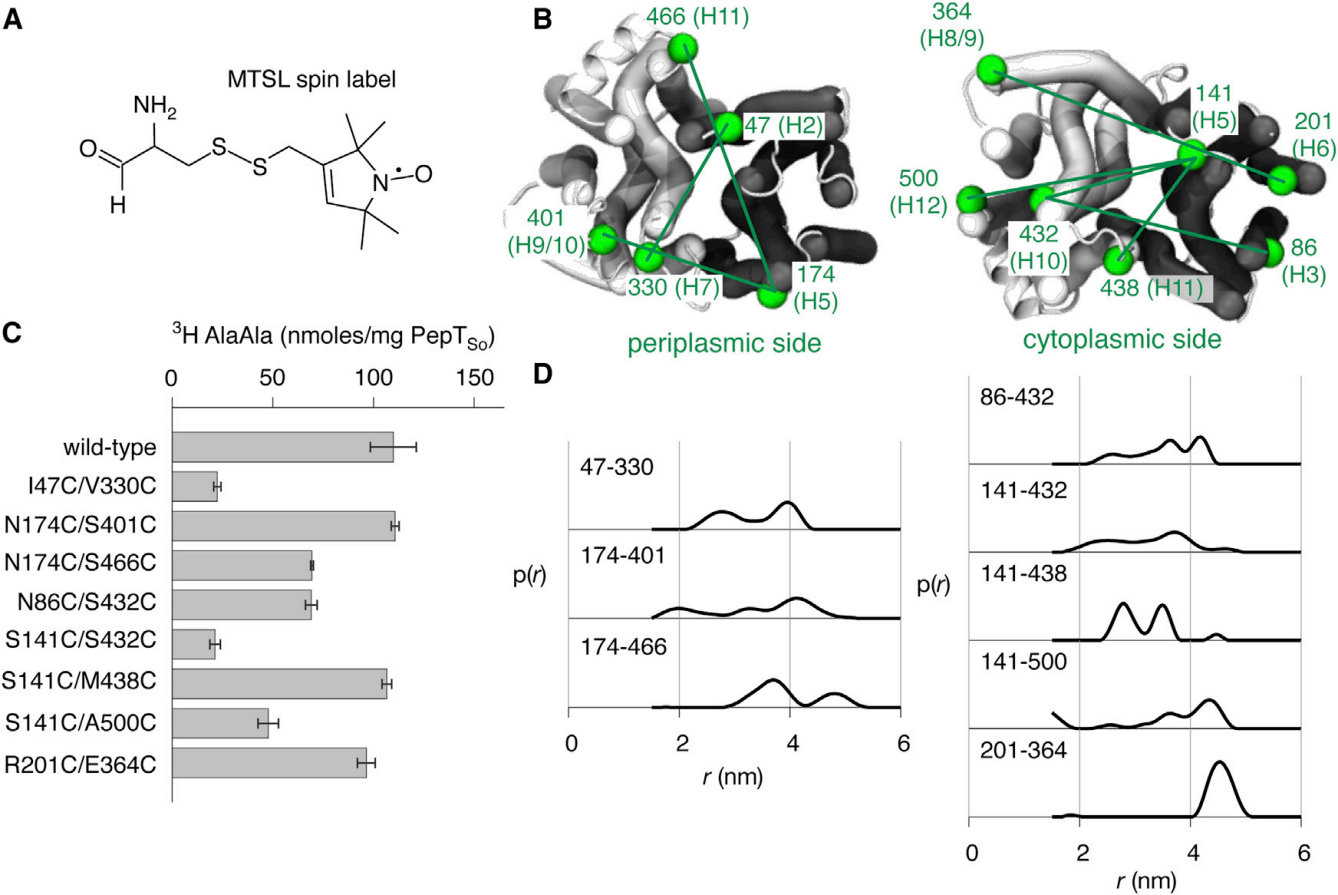
An Electron Paramagnetic Resonance Technique, DEER, Was Used to Measure Distance Distributions for Eight Pairs of Residues of PepT_So_ (A) The MTSL spin label has a flexible linker with a maximum length of 0.9 nm. (B) Three pairs of residues on the periplasmic face and five on the cytoplasmic face (both in green) of the transporter were labeled with the spin label MTSL. (C) Adding the spin labels requires pairs of cysteines to be introduced. The activity of these double mutants was checked using an uphill transport assay. This showed that while none of the mutants abolished transport, several did decrease the rate at which PepT_So_ could transport. (D) The DEER distance distributions, *p*(*r*). Error bars indicate the standard deviations from triplicate experiments.

Site-directed spin labeling combined with an electron paramagnetic resonance technique, double electron-electron resonance (DEER, also known as PELDOR, pulsed electron-electron double resonance; Reginsson and Schiemann, 2011; Mchaourab et al., 2011) is a biophysical method for determining the distance distribution between two labeled cysteines in a protein. PepT_St_, however, formed dimers in solution, complicating the interpretation of the spin-spin distances, and hence was less suitable. Eight pairs of cysteine residues were introduced into PepT_So_ and labeled with the nitroxide spin label (1-oxyl-2,2,5,5-tetramethylpyrroline-3-methyl)methanethiosulfonate (MTSL) (Figure 3A); these pairs were designed to measure three periplasmic distances and five cytoplasmic distances on the protein (Figure 3B). The transport activity of all eight double cysteine mutants was tested using a proton-coupled assay and all eight were competent at active transport, although some mutants were less active than wild-type (Figure 3C). The experimental DEER distance distributions are broad, often covering ∼30 Å, and are typically multimodal, usually with two or three distinct peaks (Figure 3D). This suggests that PepT_So_ is highly dynamic and is present in several different conformations during the experiment.

Since the MTSL label has a flexible linker, it can adopt a wide range of conformations when bound to a cysteine residue. The DEER distance distributions are hence convolutions of all the protein conformations present with all possible conformations of the spin label. Determining if a structure is consistent with the distance distributions derived from the DEER data is therefore complicated. To estimate the resulting broadening attributable to the flexibility of the MTSL linker, we mapped a rotamer library of spin labels onto each pair of residues, thereby estimating the spin-spin distance distribution that would arise from a single, specified structure (Stelzl et al., 2014; Polyhach et al., 2011). This method allows stronger inferences to be made than either simply calculating the distances between the C_α_ atoms of the labeled residues, or determining if, for a given structure, there are spin label rotamers consistent with the most likely distances as represented by the positions of the maxima observed in the DEER distance distributions (Madej et al., 2012).

Since the POT family comprises proton-coupled symporters, the apo protein can move between inward- and outward-open conformations, and hence we expect both conformations to be populated in the DEER experiment in the absence of substrate. Comparing the distance distributions predicted from the inward-open crystal structure and the repeat-swapped outward-open model with the experimental DEER data therefore provides a route to validate the PepT_So_ outward-open model. If the experimental data fit the peaks in both sets of predicted distance distributions then that would be a strong validation of the model. Not being able to explain all the features of the predicted distance distributions, discrepancies in the position or width of the peaks or variation between the different residue pairs studied would weaken the level of validation. Finally, predicted distance distributions that simply lie within the bounds of the experimental distributions but whose peaks do not exactly match the experimental DEER data would constitute a weak form of validation.

The correspondence between the experimental DEER data and the distributions predicted from the inward-open PepT_So_ structure and outward-open PepT_So_ model falls somewhere between the last two levels; taken together, the positions of all the predicted peaks do not align exactly with the peaks in the DEER data nor do the distributions explain all the features of the experimental data. The latter suggests that the protein is sampling more than two distinct conformations, which complicates the validation. The spin-spin distance distributions predicted from the experimental inward-open PepT_So_ structure lie within the bounds of the DEER distance distributions for seven of the eight pairs of residues examined (Figure S3; the exception is the 141–500 distance, which has limited overlap). Of these seven, the positions and widths of the predicted peaks align reasonably for six of the seven remaining residue pairs, the exception being the 174–401 distance. For the outward-open model the predicted spin-spin distance distributions lie within the bounds of six of the DEER distance distributions, the exceptions being the 141–500 (again) and 201–364 distances (Figure S3). We shall address why the model poorly describes the 201–364 distance later. The predicted distributions align reasonably for five of the six remaining residue pairs, the exception being the 47–330 distance. The outward-open model therefore agrees slightly less well with the experimental DEER data than the inward-open PepT_So_ crystal structure and hence is only weakly quantitatively consistent with the experimental DEER data.

It is surprising, however, that the distance distributions predicted from the inward-open crystal structure of PepT_So_ do not better describe the experimental DEER distance distributions. This suggests that our DEER-based approach would struggle to discriminate between good and poor models. There are several possible reasons for this. First, the experiments were carried out in detergent, which may have affected the dynamics of the protein. Second, our method assumes that the dynamics of the protein and the dynamics of the spin label (its rotamer states) are independent (although clashing rotamers are removed). Allowing the rotamers adopted by the MTSL spin label to be influenced by the conformation of the protein may improve the predicted distance distributions and hence the correspondence with the experimental DEER data (Roux and Islam, 2013).

### Generating Outward-Open Conformations by Molecular Dynamics Simulation

We therefore attempted a second, independent approach to producing structures of both PepT_So_ and PepT_St_ in outward-open conformations. This was to run long molecular dynamics simulations of a single copy of each protein embedded in a lipid bilayer as described in the Experimental Procedures. Three simulations, each 200 ns long, were run for each protein. Since this is comparatively short, we did not expect to see transitions in all simulations. Inspecting how the arrangement of the transmembrane helices changed suggested that, in at least one of the simulations for both proteins, there was a partial transition toward the outward-open state due, we assume, to the stochasticity of the dynamics. We then categorized and clustered the conformations produced by the simulations based on the state of the cytoplasmic and periplasmic gates.

### Determining the Conformational State of an MFS Transporter

**Figure 4:**
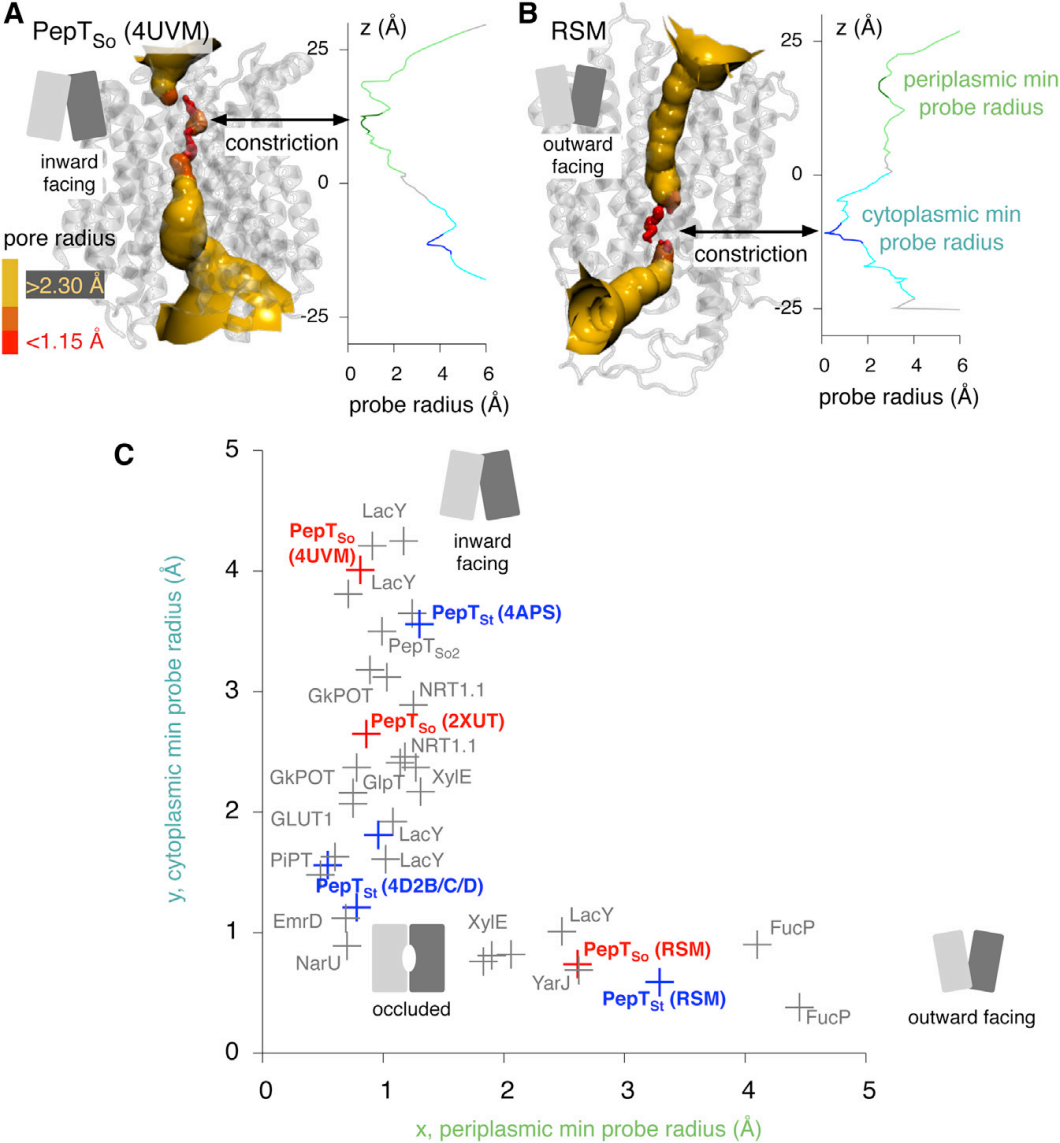
The Conformational State of an MFS Transporter Can Be Accurately Determined by Passing a Spherical Probe from One Side of the Protein Structure to the Other (A) The percolation surface through the structure of PepT_So_ is shown. This was calculated using HOLE (Smart et al., 1996) as described in the Experimental Procedures. The surface is colored according to the maximum radius of the spherical probe; less than 1.15 Å is colored red, greater than 2.30 Å yellow and, in between, orange. The pore profile (the variation in the maximum radius of a spherical probe as a function of *z*) can be used to identify constrictions. The maximum radius of a probe that can pass any constriction is estimated as the average of the probe radius over a window 4 Å wide centered on the constriction (i.e. the minimum value). The periplasmic and cytoplasmic gate regions in the pore profile are colored light green and cyan and the 4 Å windows colored dark green and dark blue, respectively. (B) The same analysis repeated on the outward-open model of PepT_So_. RSM, repeat-swapped models. (C) This analysis has also been repeated for PepT_St_ and all other known MFS structures (Figure S4). The coordinates of the crystal structures and outward-open models of both PepT_So_ and PepT_St_ are shown in red and blue, respectively.

Although a simple distance-based method for determining the conformational state of a structure of an MFS transporter has been recently proposed (Stelzl et al., 2014), it assumes which helices characterize the state of a transporter. We have extended the ideas of Stelzl et al. (2014) by considering all possible permutations of helices and thereby determining a priori which helices form the cytoplasmic and periplasmic gates of MFS transporters. To provide a reference data set we started by determining the maximum radius of a spherical probe that can be accommodated in the protein along the z axis, i.e. as the probe is moved from the cytoplasm, through the central cavity, and into the periplasm. The resulting pore profiles confirm that the central cavity in the new structure of PepT_So_ is accessible to the cytoplasm but inaccessible to the periplasm, hence the structure can be described as inward open (Figure 4A). Likewise, the central cavity of the model built using the repeat-swapping method is accessible to the periplasm but inaccessible to the cytoplasm (Figure 4B) and so can be described as outward open. Similar results were also obtained for PepT_St_ (Figure S4).

The same procedure was then applied to all currently known structures of MFS transport proteins (Figure S4), and the minimum value of the radius in both gate regions was determined. Plotting the minimum probe radius of the cytoplasmic gate against the same quantity for the periplasmic gate (Figure 4C) elegantly quantifies the conformational state of all known MFS transporter structures. As expected, the upper right quadrant in Figure 4C is empty since there are no crystal structures with both gates open; the coordinates instead describe an L-shaped locus. The MFS transporters in the top left quadrant have an open cytoplasmic gate but a closed periplasmic gate, and are therefore inward facing. This relationship is reversed in the bottom right quadrant (these are the outward-facing structures) while those in the bottom left quadrant are occluded. Analyzing the pore radius profiles in this way provides an intuitive and accurate way to characterize the conformation of an MFS transporter and has allowed us to assign, rigorously and without bias, each of the known MFS structures to a specific state. This method is, however, both time consuming to apply to molecular dynamics trajectories and does not allow the helices forming the gates to be identified. To address these problems we shall develop a simple geometrical metric based on the minimum distance between the tips of the transmembrane helices in the protein (Stelzl et al., 2014).

**Figure 5:**
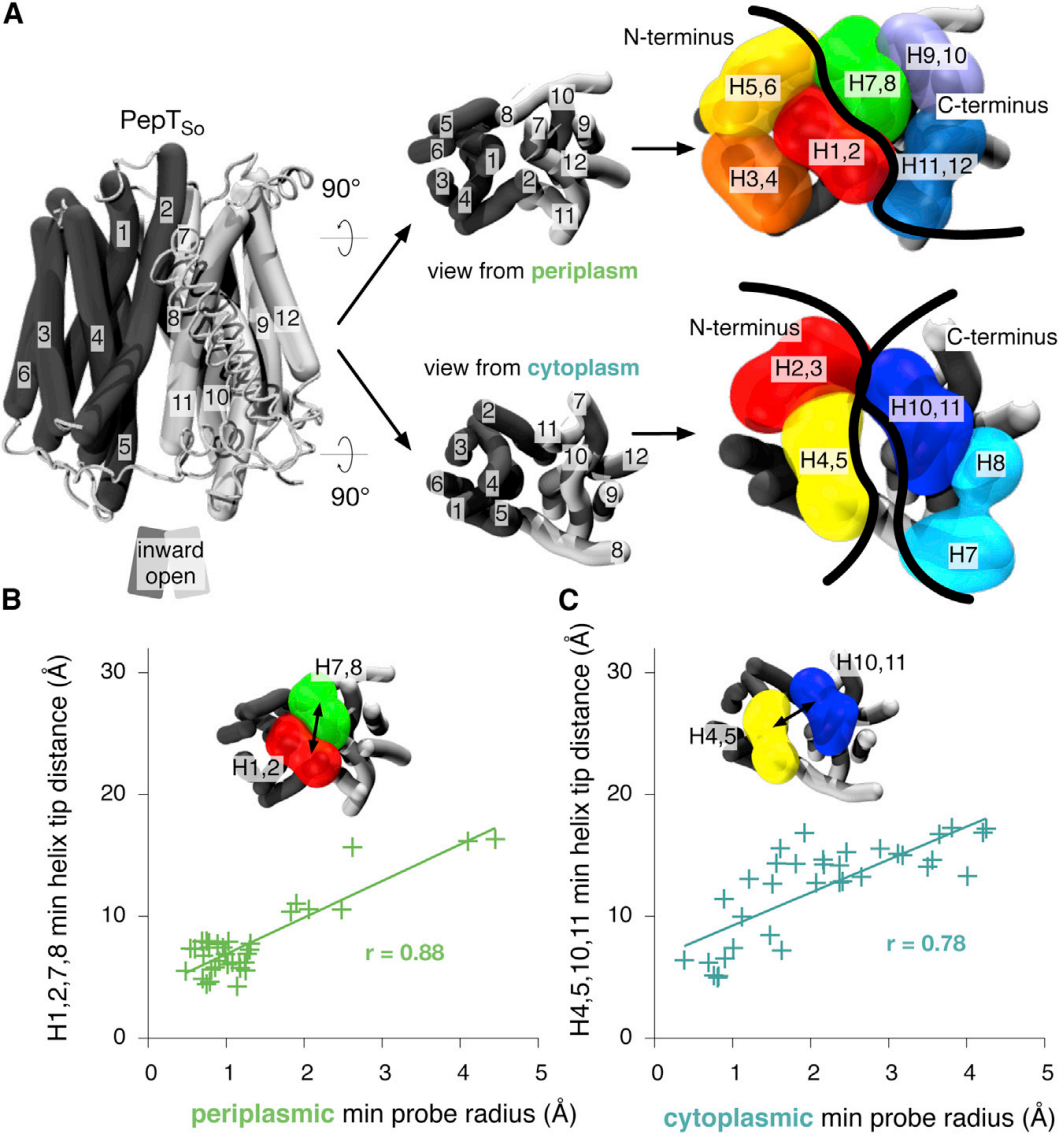
The Minimum C_α_-C_α_ Distance between the Tips of H1 & H2 and H7 & H8 Correlates Best with the State of the Periplasmic Gate and the Minimum C_α_-C_α_ Distance between the Tips of H4 & H5 and H10 & H11 Correlates Best with the State of the Cytoplasmic Gate (A) There are three possible contiguous helix pairs in each half of the protein on the periplasmic side and only two possible contiguous helix pairs in each half of the protein on the cytoplasmic side. To determine which pairs of helix tip pairs constitute the gates of the transporter, the minimum C_α_-C_α_ distance between the tips of all possible helix tip pairs was calculated. (B) The distance between H1 & H2 and H7 & H8 correlated most closely with the state of the periplasmic gate, as determined by HOLE (r = 0.88). (C) The distance between H4 & H5 and H10 & H11 correlated most closely with the state of the cytoplasmic gate, as determined by HOLE (r = 0.78).

We assume that both gates in MFS transporters are formed by the tips of two contiguous pairs of transmembrane helices coming together, one pair from each half of the protein. This is consistent with previous suggestions about which helices contribute to one or other of the gates (Huang et al., 2003; Solcan et al., 2012). Since each half of the transporter is made up of six transmembrane helices and the N terminus of the protein is found in the cytoplasm, there are three helix pairs in each half of the transporter that could contribute to the periplasmic gate (Figure 5A: H1 & H2, H3 & H4, H5 & H6 and H7 & H8, H9 & H10, H11 & H12) and two helix pairs in each half of the transporter that could contribute to the cytoplasmic gate (H2 & H3, H4 & H5 and H8 & H9, H10 & H11). We only considered the C_α_ atoms of the tip of each helix, defined as the first or last ten residues of each helix on the cytoplasmic or periplasmic side of the protein. The minimum distance between each set of helix tip pairs was then calculated. This was repeated for all nine combinations of periplasmic helix tip pairs and four combinations of cytoplasmic helix tip pairs for all known MFS transporter structures. We then examined the correlation between these sets of distances and the previously determined minimum probe radius that can pass through each of the gates (Figure S5). The state of the periplasmic gate correlates best with the minimum distance between the tips of helix pairs H1 & H2 and H7 & H8 (r = 0.88, Figure 5B; Figure S5A), while the state of the cytoplasmic gate correlates well with the minimum distance between the tips of H4 & H5 and H10 & H11 (r = 0.78, Figure 5C; Figure S5B), thus suggesting that not only do these helices form the periplasmic and cytoplasmic gates but also that these simple distance-based metrics accurately capture the state of these gates. It is not surprising that these particular four helix pairs make up the periplasmic and cytoplasmic gates of MFS transporters since they are, in both cases, closest to the axis of symmetry that divides the N- from the C-terminal halves in any MFS transporter (Figure 5A).

**Figure 6:**
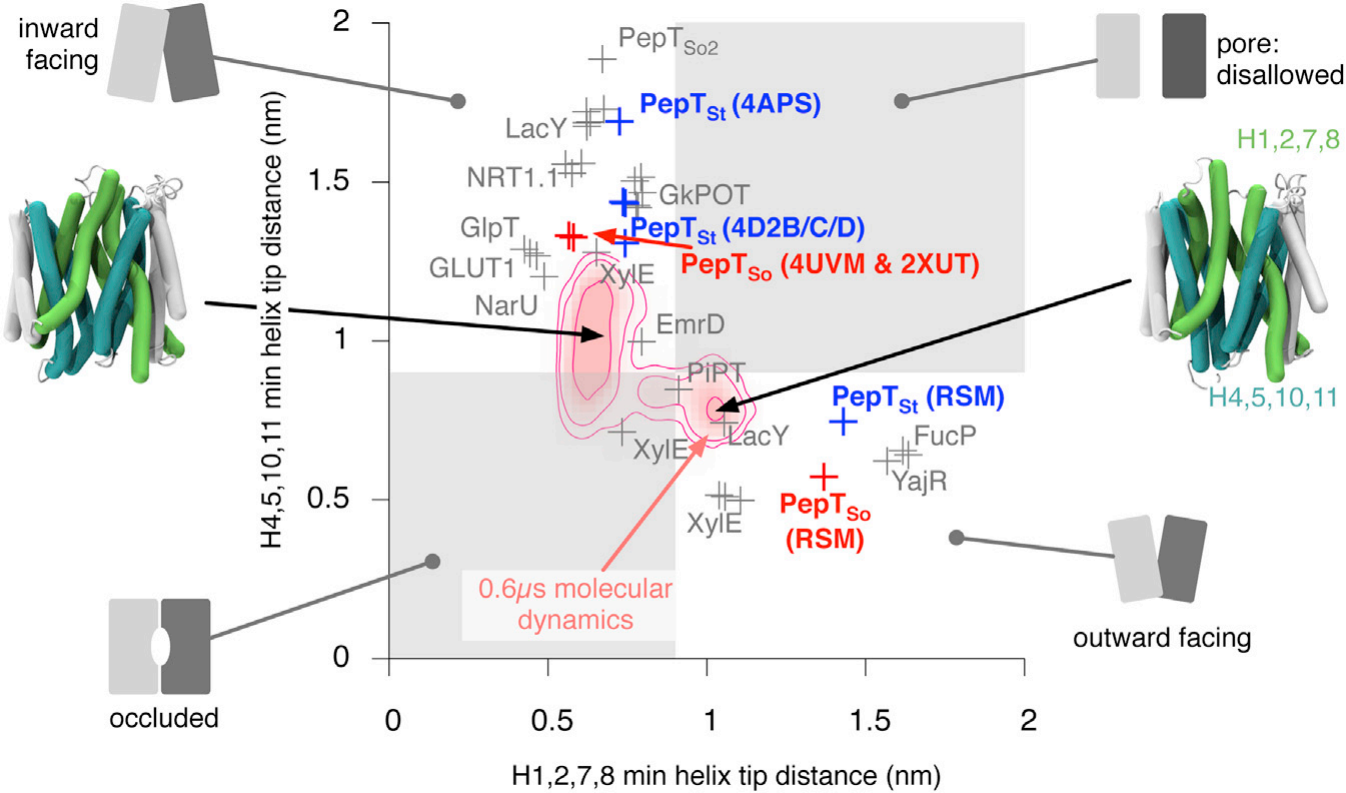
During the Molecular Dynamics Simulations PepT_So_ Explores Inward-Facing, Occluded, and Some Partially Outward-Facing Conformations, as Defined by the Minimum Distance between the C_α_ Atoms of the Relevant Pairs of Helix Tips The density of states explored during the simulations starting from the 2XUT PepT_So_ structures are plotted in pink, and two representative inward-facing and outward-facing structures are shown. The coordinates of known MFS structures are plotted to provide some context, and the different quadrants of the coordinate space are labeled. The coordinates of the PepT_So_ and PepT_St_ crystal structures and repeat-swapped models (RSM) are labeled in red and blue, respectively. The results from the PepT_St_ simulations can be found in Figure S6.

We then examined how the conformations of PepT_So_ and PepT_St_ changed during the molecular dynamics simulations by projecting the density of states onto the 2D space defined by our new distance-based metrics (Figure 6; Figure S6). Both proteins sample inward-facing, occluded, and outward-facing conformations during each set of three simulations. We defined any conformation that has both distances less than 9 Å as being occluded and any conformation with the periplasmic distance ≥9 Å and the cytoplasmic distance <9 Å as being outward open (and the other way round for inward open). This allowed us to classify the ensemble of structures generated during the simulations as either outward facing, inward facing, or occluded. For both proteins, one of the three simulations explored parts of the outward-open region. The C-terminal half of both proteins was found to be more dynamic than the N-terminal half (Table S1), consistent with the differences between the crystal structures of PepT_So_ and PepT_St_. This pattern continued when the repeat-swap units were analyzed, with C and D being more dynamic than repeat units A and B. Examining the individual transmembrane helices showed that within each repeat unit, the third helix was typically less dynamic than the first two. Each conformation generated by the simulations was then analyzed for the presence of salt bridges.

### Salt Bridges Stabilize the Intracellular Gate in the POT Family

**Figure 7:**
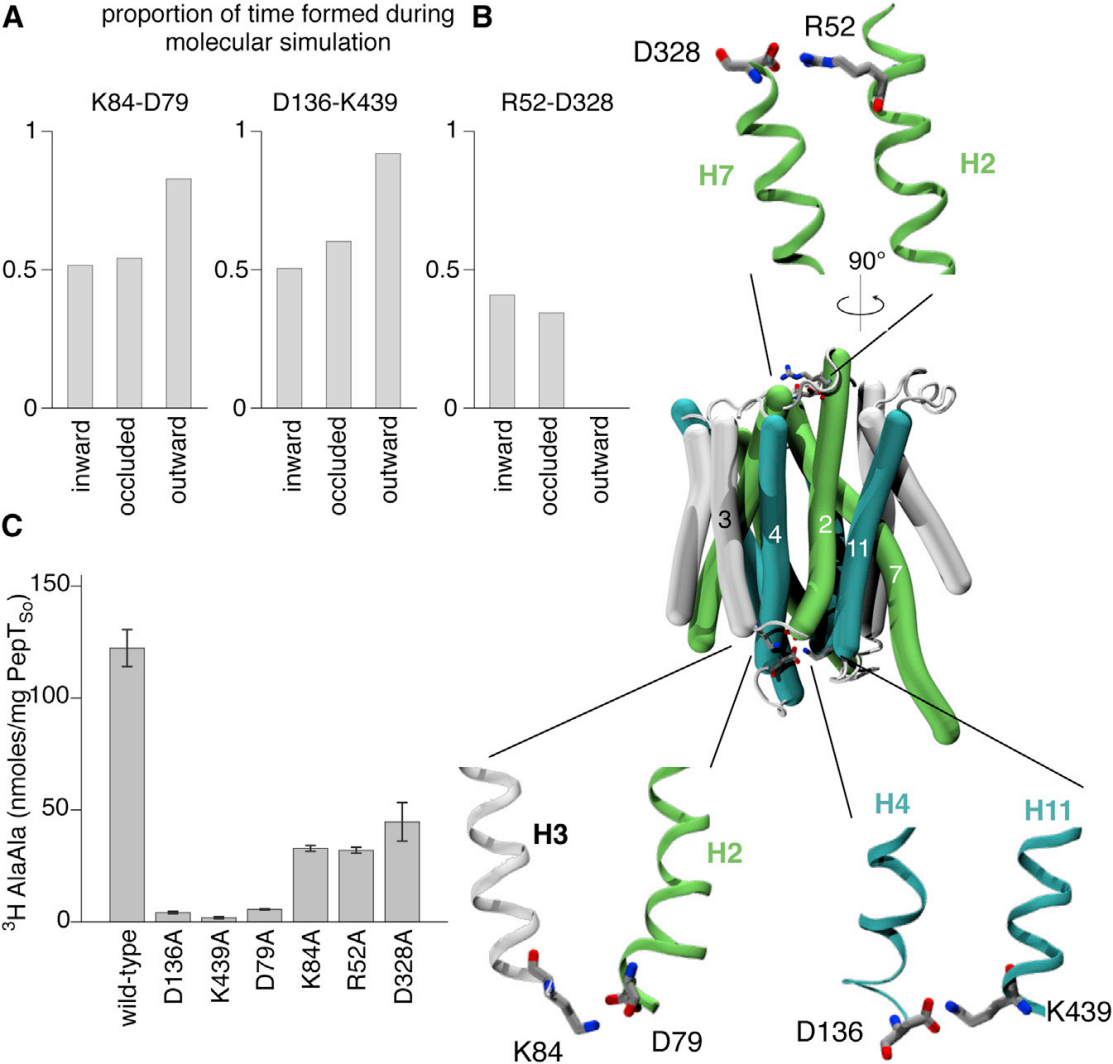
Two Salt Bridges Are Predicted to Stabilize Outward-Facing Conformations of PepT_So_ (A) The ensemble of conformations produced by the molecular dynamics simulations were analyzed for salt bridges and the conformation classified as defined in Figure 6. Seven salt bridges in total were found. The three whose propensities are a function of the conformation of the transporter, and therefore may stabilize one of more conformational states, are shown here; the others are described in the Supplemental Information (Figure S7). (B) Two of the salt bridges (K84-D79 and D136-K439) are found on the cytoplasmic side of PepT_So_, while the third (R52-D328) occurs on the periplasmic side. Since these are found in different conformations, the pull-out figures are taken from different parts of the molecular dynamics trajectories. (C) All the alanine mutants were either inactive in transport or had significantly reduced function. Error bars indicate the standard deviations from triplicate experiments.

Seven salt bridges were identified in the simulations of PepT_So_ (Figure 7; Figure S7). Two interactions (D136-K439 and K84-D79) were predicted to stabilize outward-facing conformations of PepT_So_, one (R52-D328) was predicted to form in inward-facing and occluded conformations (Figure 7A), and the remaining four are discussed in the legend of Figure S7. The residues in the first two salt bridges are conserved in mammalian members of the POT family, but only the second salt bridge has the potential to form in PepT_St_. The side chains of D136 and K439 are pointing away from one another in our outward-open model of PepT_So_ and their C_α_ atoms are 1.5 Å further apart than when a salt bridge has formed in the simulations. The repeat-swapped model of PepT_So_ does not therefore predict the D136-K439 salt bridge, although only a small motion is required for it to form. Since the residues of the second salt bridge (K84-D79) are close in sequence, and therefore the distance between them is always small, it is not possible to say if the outward-open model predicts the K84-D79 interaction. Mutating any of these four residues to alanine abolishes transport or, in the case of K84A, reduces it significantly, which is consistent with (but does not prove) the hypothesis that these salt bridges stabilize outward-facing conformations of the POT family.

Finally, let us consider the putative R52-D328 interaction. Although these two residues are not interacting with one another in the inward-facing structure of PepT_So_, again only comparatively small motions would be required to bring this about. These residues are not, however, conserved within the SLC15 family and mutating either residue to alanine merely reduces transport, suggesting that even if this interaction does stabilize inward-facing conformations, it is not essential and is only found in bacterial members of the POT family. Analysis of the PepT_St_ simulations identified five salt bridges (Figure S7), two of which (R33-E300 and R53-E312) have been previously suggested to stabilize inward-facing conformations (Solcan et al., 2012) and two of which (K126-E25 and K126-E22) are equivalent to those seen in PepT_So_.

### A Proline-Induced Kink in H8 Is Required for Transport in the POT Family

**Figure 8:**
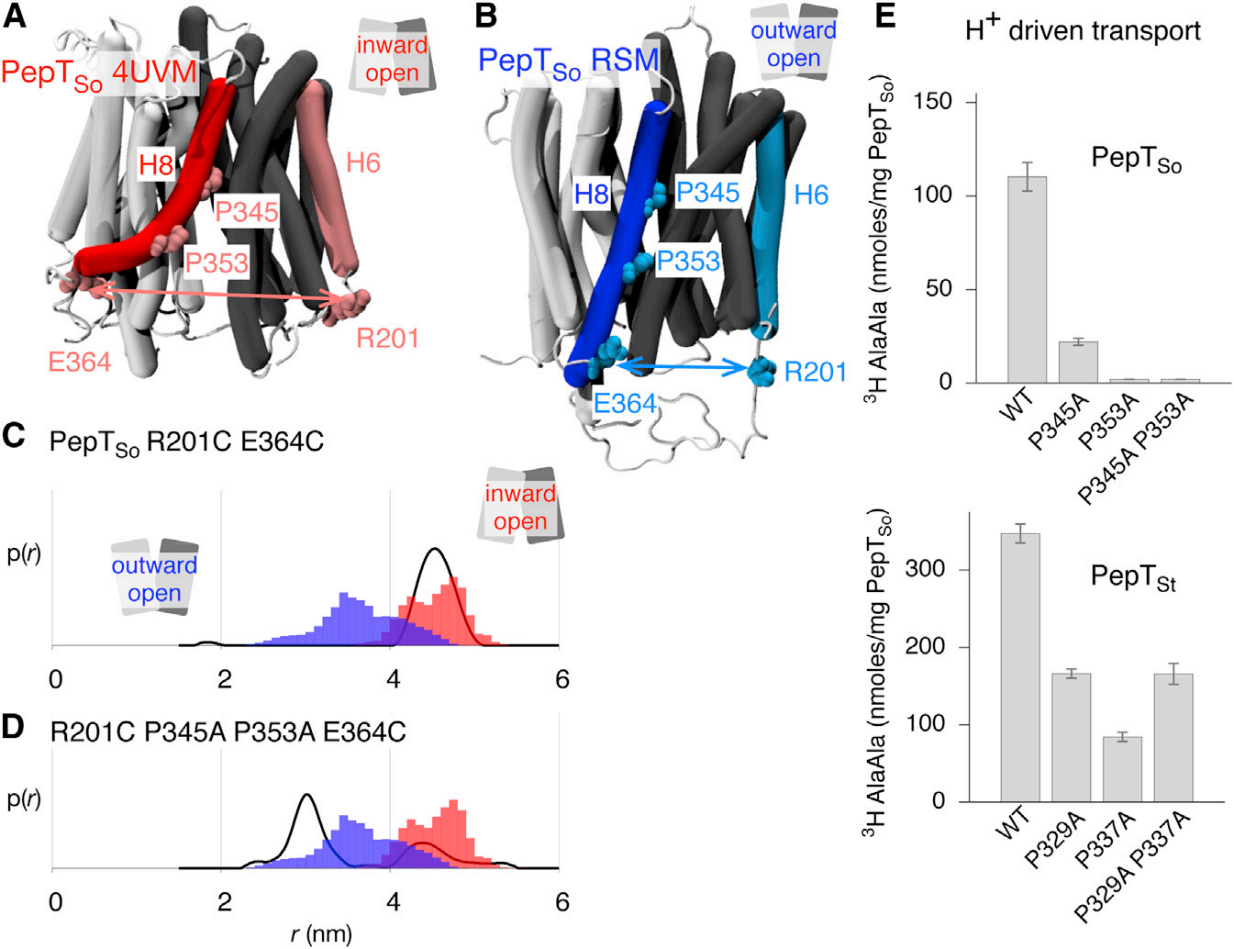
The Kink Produced by the Conserved Prolines in H8 Is Important for Transport (A) In the inward-occluded experimental structure of PepT_So_, H8 (in red) is kinked because of two prolines, P345 and P353 (in pink). We measured the relative motion of the C-terminal ends of H8 and H6 (pink) by attaching MTSL spin labels to the E364C R201C mutant of PepT_So_. (B) The same features are highlighted in blue on the outward-open model of PepT_So_, demonstrating that this model predicts a shorter distance between positions 201 and 364. (C) There is moderate overlap between the R201C E364C spin-spin distance distributions measured experimentally (black line) and those predicted from the inward-occluded crystal structure of PepT_So_ (filled red bars). The outward-open model instead predicts a shorter distance between the ends of H6 and H8 (filled blue bars). (D) Mutating both prolines to alanine results in a more complex spin-spin distance distribution. We suggest that H8 in the R201C E364C P345A P353A is straighter than wild-type. Consistent with this, there is now reasonable agreement between the spin-spin distance distributions measured experimentally (black line) and those predicted from the model of the outward-facing conformation (filled blue bars). (E) Mutating either or both prolines in PepT_So_ or PepT_St_ either reduces or abolishes proton-driven active transport. Error bars indicate the standard deviations from triplicate experiments.

We previously noted the poor agreement between the DEER E201C-R364C distance distributions and our predictions from the outward-facing PepT_So_ model (Figure S3). This pair of residues reports the relative motions of H6 and H8, respectively (Figure 8A), which are part of repeat units B and C, respectively. A major structural difference between repeat units C and D is the kink in H8, which is absent in its symmetry-related partner, H11. Examining the structure of PepT_So_ suggests that the kink in H8 is due to two prolines, P345 and P353 (P329 and P345 in PepT_St_), the latter being highly conserved across the POT family (Figure S8). Prolines are known to favor kinks in transmembrane helices (Fowler and Sansom, 2013). Mutating both prolines to alanine led to an altered spin-spin distance distribution in PepT_So_ with two peaks, one at a position similar to that of the single peak observed in wild-type, and another, more dominant, peak at a shorter distance (Figure 8D; Figure S3). This second peak overlaps with the distance distribution predicted from the outward-facing model and is likely due to a straightening of H8 caused by the removal of the two proline residues, resulting in a decrease in the distance between the intracellular ends of H6 and H8. We propose that the anomalously short distance predicted by the outward-open model of PepT_So_ is the result of the repeat-swapping process making H8 too straight. To support this hypothesis, mutating the first proline in either PepT_So_ (P345A) or PepT_St_ (P329A) reduced proton-driven transport (Figure 8E). The effect was more pronounced when the second, more conserved proline was mutated; the P353A PepT_So_ mutant abolished active transport and the P345A PepT_St_ mutant had only 20% of the level of transport activity of the wild-type. The PepT_So_ double mutant had no detectable transport activity, whereas the PepT_St_ double mutant had activity similar to that of the P329A mutant.

What about helix H11? It is approximately straight in the crystal structures but kinked in the outward-open models, since its sequence is threaded onto the structure of H8. The 174–466 and 141–438 residue pairs report the relative motions of helices H5 and H11 (Figure 3B). The distance distributions predicted for both pairs of residues appear to agree moderately well with the DEER data (Figure S3), although neither is shifted significantly by the conformational change. If the kink in H11 is an artifact of the repeat-swapping process then it is likely that this will bias both these predicted distance distributions. It is probable, however, that H11 bends to some extent as it has a central glycine (Gly453 in PepT_So_, Gly434 in PepT_St_), which is conserved across the POT family (Figure S8).

Our results suggest that (1) proline-induced kinks in transmembrane helices are important for the function of POT family transporters (Madej et al., 2012), (2) the repeat-swapping method captures the internal dynamics of a domain best when the repeat units are not too dissimilar to one another, and (3) taking into account the presence (or absence) of kink-forming residues in symmetry-related helices could further improve the repeat-swapping method.

## Discussion

Alternating access within secondary active transporters is currently understood to occur through the formation of three distinct sets of conformations: the outward-facing, occluded, and inward-facing states (Yan, 2013). As their names suggest, the three states are differentiated by whether gates permit or block access to the central cavity from either side (or both sides) of the membrane. We have studied outward-facing conformations of two members of the POT family using two independent methods; we built outward-open models of PepT_So_ and PepT_St_ using the repeat-swapping method and also ran molecular dynamics simulations starting from inward-facing crystal structures. The PepT_So_ outward-open model is only weakly consistent with a set of eight spin-spin distances measured by DEER spectroscopy. The DEER data suggest that apo PepT_So_ samples more than two conformations, which complicates the interpretation.

### A Double Scissor-Switch Mode of Gating within the POT Family

**Figure 9:**
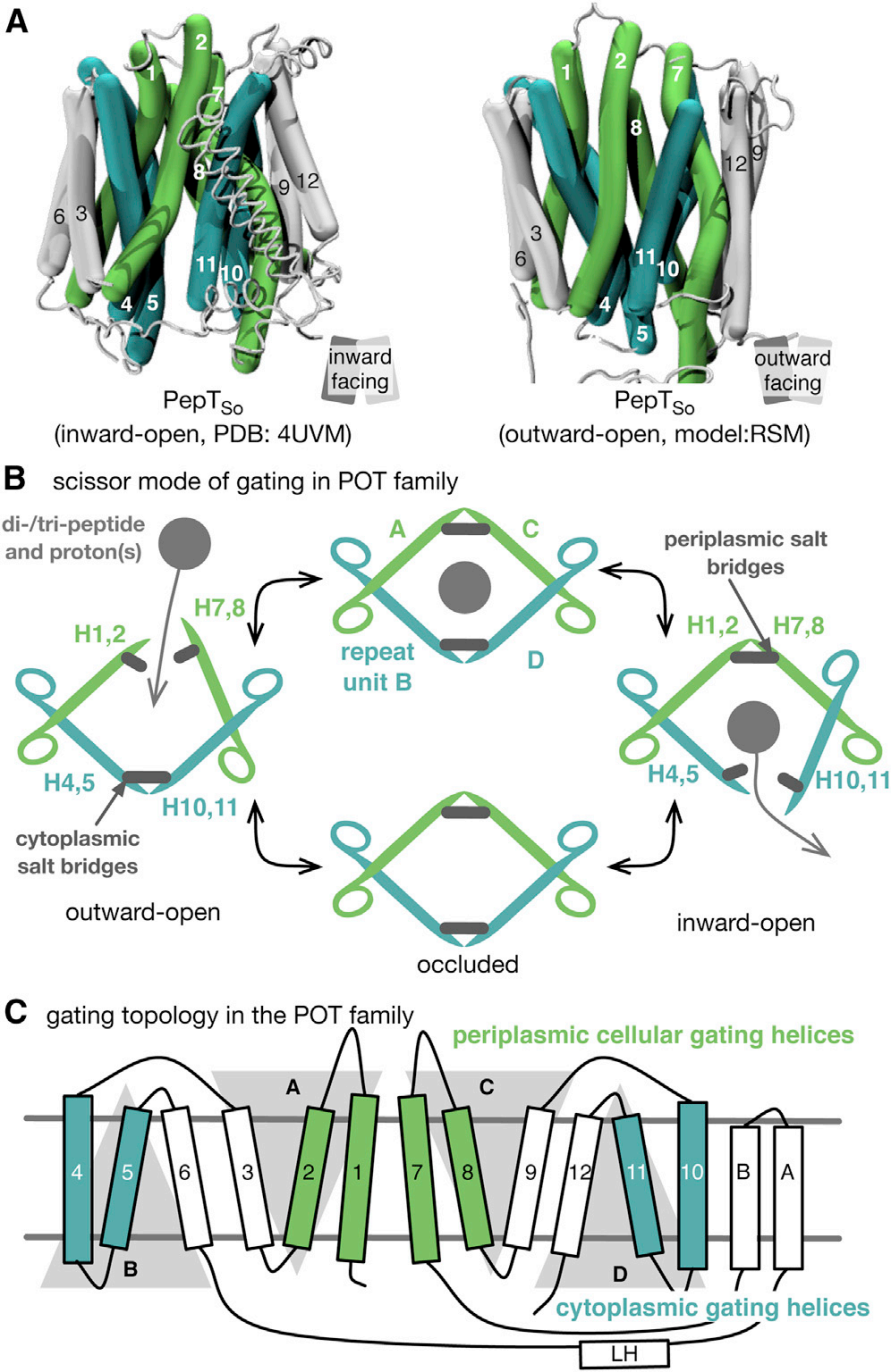
The Periplasmic and Cytoplasmic Gates of Proton Oligopeptide Transporters Are Formed from Two Bundles of Four α helices (A) The periplasmic gate is formed by H1, H2, H7, and H8 (colored green) and the cytoplasmic gate is formed by H4, H5, H10, and H11 (cyan). (B) The motion of the helices is described by a pair of scissors; this captures the concerted scissoring movement of the two bundles of helices and the increased motion of H7–H12, compared with H1–H6. Also shown are schematic salt bridges that stabilize the closed gates. (C) The first two helices from each repeat unit therefore contribute to either the periplasmic gate (green) or the cytoplasmic gate (cyan). The third helix in each repeat-swapped unit is located at the periphery, and does not play a direct role in gating the transporter.

Our experiments, models, and simulations suggest that in the POT family, the first six helices are less dynamic than the last six, and that the cytoplasmic gate is formed by helices H4 and H5 (from repeat unit B) packing against H10 and H11 (from repeat unit D, Figure 9A). In PepT_So_, this gate is apparently stabilized by salt bridges between Asp136 (H4) and Lys439 (H11) and Asp79 (H2) and Lys84 (H3) (Figure 7). Upon binding a peptide and proton(s), the periplasmic gate closes, characterized by the movement of helices H7 and H8 toward H1 and H2. Transport appears to require the kink in helix H8 formed by two conserved proline residues to be maintained. The salt bridges stabilizing the cytoplasmic gate then break, and helices H10 and H11 swing away from H4 and H5, opening the gate. The four helices not involved in either gate, which we call the scaffold helices (H3, H6, H9, H12), are less mobile and provide a platform against which the gating helices can move.

It is illuminating to consider a simple model whereby PepT_So_ or PepT_St_ are described by two pairs of scissors, with each pair representing either the N- or C-terminal half of the protein and each blade in a pair of scissors embodying one of the four repeat units (Figure 9B). The gating motions described above are hence analogous to both pairs of scissors opening and closing in a concerted manner. The hinges where the blades slide past one another are also likely to be important (Yaffe et al., 2013). Crucially, there is asymmetry in the relative magnitudes of how much each pair of scissors moves: the blades of the C-terminal scissors move more than the blades of the N-terminal scissors. This observation is consistent with a previous analysis comparing the asymmetric PepT_So_ structure with structures of the lactose permease, LacY (Newstead et al., 2011). Our scissor analogy also extends the “rocker-switch” mechanism (Huang et al., 2003; Law et al., 2008) by including not just the symmetries between the N- and C-terminal halves of POT family transporters but also the recently identified three-helical inverted-topology repeats (Radestock and Forrest, 2011). This double scissor mode of gating links the structural organization and symmetries within the MFS fold to the currently available structural and biochemical data for the POT family and hence provides a working mechanism for proton-coupled peptide transport across the membrane.

### The Gating Topology of MFS Transporters

Our conclusion that the periplasmic gate comprises H1, H2, H7, and H8 and the cytoplasmic gate is made from H4, H5, H10, and H11 is consistent with all 34 known MFS structures (Figure S9) and is therefore likely to apply across the superfamily. Subsets of these helices have been previously proposed to form one or the other of the gates (Huang et al., 2003; Newstead et al., 2011; Solcan et al., 2012). We built on ideas introduced by Stelzl et al. (2014) to systematically demonstrate that the minimum distance between the tips of these helices correlates with the minimum radius of a spherical probe able to pass through the gate (Figure 5). This simple distance-based metric is also likely to apply across the whole MFS superfamily.

When we map these eight helices back onto the transmembrane topology, we find a surprising and pleasing symmetry (Figure 9C); the first two helices of each of the four repeat units are found to form half of one of the two gates. A picture emerges whereby H1 and H2 from repeat unit A and H7 and H8 from repeat unit C come together to form the periplasmic gate (and likewise the cytoplasmic gate is formed by H4 and H5 from repeat unit B and H10 and H11 from repeat unit D). By linking the concept of inverted-topology repeat units with our observation of which helices form the gates, we are therefore able to suggest a putative gating topology of the MFS superfamily (Figure 9C).

Some of our observations, however, cannot be extended from the POT family to the wider MFS superfamily, reinforcing the view that individual families have evolved unique transport mechanisms. For example, we designed the DEER experiments so that five of the distances we studied were equivalent to five distances previously studied in the lactose permease, LacY (Smirnova et al., 2007). Unexpectedly, the spin-spin distances for PepT_So_ and LacY (Figure S9) were very different, suggesting that, despite both being bacterial proton-coupled symporters with hydrophilic substrates, the precise dynamics of these two MFS transporters are different. Likewise, it is unlikely that any of the salt bridges we have predicted and tested will be conserved outside the POT family, although the general concept may hold as similar stabilizing interactions have been proposed for VMAT2, another MFS transporter (Yaffe et al., 2013). While it is also likely that the role of the prolines in H8 is specific to the POT family, Brandl and Deber (1986) noticed nearly 30 years ago that prolines are overrepresented in the transmembrane α helices of transport proteins, the largest class of which are the MFS transporters. We anticipate that future studies will unravel the role of prolines and kinked helices in the functioning of MFS transporters.

## Experimental Procedures

The key methods are summarized here; detailed descriptions can be found in the Supplemental Experimental Procedures.

### Building the Repeat-Swapped Models

Preliminary pairwise sequence alignment between the two halves of the PepT_So_ and PepT_St_ sequences were constructed by superposing the structural repeats of each protein onto one another as described elsewhere (Radestock and Forrest, 2011). The additional helices present in both proteins, HA and HB, are not part of any repeat unit and so were omitted from all model building. Both preliminary alignments were then adjusted manually to remove gaps in the TM helices, and the sequences of individual helices were shifted to improve the sequence conservation. Using these sequence alignments the repeat-swapped models of PepT_So_ and PepT_St_ were constructed using Modeller 9.7 (Sali and Blundell, 1993). The inward-open PepT_So_ (PDB: 4UVM) and PepT_St_ (PDB: 4APS) crystal structures were used as templates (Solcan et al., 2012). The 100 PepT_So_ and PepT_St_ models with the lowest scores were refined further. The two structures with the lowest Modeller (DOPE) scores were selected to be the representative, one for each protein. Both repeat-swapped models are available in the Supplemental Information.

### Protein Purification and Crystallization, and Data Collection and Processing

Wild-type and mutant PepT_So_ were purified to homogeneity (Newstead et al., 2011). Crystals were prepared as described previously (Lyons et al., 2014). X-Ray diffraction data were collected on the I24 beamline at the Diamond Light Source, Oxford, UK. Molecular replacement search models were prepared from the inward-occluded PepT_So_ model (PDB: 2XUT). More detail is given in the Supplemental Information, and the data collection and refinement statistics are shown in Table 1.

### Transport Assay

Both PepT_So_ and PepT_St_ were reconstituted into *Escherichia coli* total lipids with egg PC liposomes and assayed using a proton-driven system as previously described (Solcan et al., 2012).

### DEER

Double cysteine mutants labeled with MTSL were prepared as described in the Supplemental Information. MTSL was obtained from Toronto Research Chemicals (North York, Canada). All measurements were carried out on a Bruker Elexsys 680 at X-band (∼9.5 GHz) between 50 and 80 K using an overcoupled (Q ≈ 100) 3 mm ER4118X-MS3 resonator. Both four-pulse (4p) and three-pulse (3p) DEER experiments were carried out. Data were processed and analyzed using DeerAnalysis2011 (Jeschke et al., 2006).

### Molecular Dynamics Simulations

Molecular dynamics simulations of chain A from the experimental structure of apo PepT_So_ (PDB ID 2XUT) were carried out as described previously by Newstead et al. (2011).

## Author Contributions

P.W.F., L.F., and S.N. designed the experiments. N.S., J.K., and S.N. made the protein, J.L. and M.C. solved the structure of PepT_So_, M.O.-R., P.M.D., and A.W. performed the DEER experiments, and N.S. and S.N. did the transport assays. S.R. and P.W.F. built the models. P.W.F. ran the simulations. P.W.F., L.F., and S.N. wrote the paper.

## Figures and Tables

**Table 1:** Data Collection and Refinement Statistics for the Inward-Open PepT_So_ Structure (PDB ID 4UVM)

	PepT_So_ (PDB ID 4UVM)
**Data Collection**	

Space group	P4_1_2_1_2
Cell dimensions (Å)	*a* = 86.83
	*b* = 86.83
	*c* = 219.82
	*α* = *β* = *γ* = 90°
Wavelength (Å)^a^	0.9686
Resolution (Å)^a^	59.13–3.0 (3.08–3.00)
No. of measured reflections^a^	84,096 (6,186)
No. of unique reflections^a^	17,156 (1,230)
*R*_merge_ (%)^a^	8.0 (83.0)
*R*_pim_^a^ (%)	4.4 (44.9)
llσl^a^	13.0 (1.9)
Completeness (%)^a^	97.5 (98.2)
Redundancy	4.9^a^ (5.0)
No. of crystals	1

**Refinement**	

Resolution (Å)	59.13–3.00
*R*_work_/*R*_free_ (%)	22.03/25.80
No. of atoms	
Protein	3921
Lipid	110
Water	17
B factors (Å^2^)	
Protein	74.167
Lipid	81.71
Water	68.11
Root-mean-square deviations	
Bond lengths (Å)	0.03
Bond angles (°)	0.771

a Highest-resolution shell is shown in parentheses.

## Accession Numbers

The PDB accession number for the PepTSo structure reported in this paper is 4UVM.

## Supplemental Information

Document S1. Supplemental Experimental Procedures, Figures S1–S11, and Table S1

Document S2. peptso-outward-rsm.pdb

Document S3. peptst-outward-rsm.pdb

Document S4. Article plus Supplemental Information
