# Supplementary material for: Gating Topology of the Proton-Coupled Oligopeptide Symporters
Source: Structure. 2015 Feb 3;23(2):290–301. doi: 10.1016/j.str.2014.12.012 (PMC4321885; doi:10.1016/j.str.2014.12.012)
Supplement: Document S1. Supplemental Experimental Procedures, Figures S1–S11, and Table S1 [file mmc1.pdf]

Structure, Volume *23*

Supplemental Information

Gating Topology of the Proton-Coupled Oligopeptide Symporters

Philip W. Fowler, Marcella Orwick-Rydmark, Sebastian Radestock, Nicolae Solcan, Patricia M. Dijkman, Joseph A. Lyons, Jane Kwok, Martin Caffrey, Anthony Watts, Lucy R. Forrest, and Simon Newstead

Contents

1 Supplemental Experimental Procedures
1.1 Materials.
1.2 Constructing the preliminary sequence alignments.
1.3 Building the repeat-swapped models.
1.4 Protein purification and crystallization.
1.5 Data collection and processing.
1.6 Structure solution and refinement
1.7 Transport assay
1.8 Preparation of $PepT_{So}$ double-cysteine mutants.
1.9 Spin-labeling protocol.
1.10 Pulsed DEER spectroscopy.
1.11 Least squares fit method for combining 3p and 4p DEER.
1.12 Determining the conformational state of MFS transporters.
1.13 Molecular dynamics simulations.
1.14 Mapping MTSL spin labels onto structures and trajectories.

2 Supplemental Figures and Tables

List of Tables

S1 The C-terminal halves of $PepT_{So}$ & $PepT_{St}$ are more dynamic than their N-terminal halves

List of Figures

S1 Major Facilitator Superfamily transporters comprise two bundles of six helices
S2 Novel features identified in the new structure of $PepT_{So}$.
S3 Validating the outward-facing model of $PepT_{So}$ against the results of the DEER experiments
S4 The percolation surfaces and pore profiles for all known structures of MFS transport proteins
S5 The periplamic and cytoplasmic gates of MFS transporters are formed by helices H1, H2, H7 & H8 and H4, H5, H10 & H11, respectively.
S6 During the molecular dynamics simulations $PepT_{St}$ explores inward-inward and some partially outward-facing conformations.
S7 Salt bridges are predicted to stabilise different conformations of $PepT_{So}$ and $PepT_{St}$.
S8 Multiple sequence alignment showing $PepT_{So}$ and $PepT_{St}$ are homologous to human PepT1.
S9 $PepT_{So}$ and LacY have different dynamics
S10 Wildtype $PepT_{So}$ aligned onto $PepT_{So}$ with its alternating repeats swapped.
S11 Wildtype $PepT_{St}$ aligned onto $PepT_{St}$ with its alternating repeats swapped.

1 Supplemental Experimental Procedures

1.1 Materials.

(1-oxyl-2,2,5,5-tetramethylpyrroline-3-methyl)- methanethiosulfonate (MTSL) was obtained from Toronto Research Chemicals (North York, Canada).

1.2 Constructing the preliminary sequence alignments.

Preliminary pair-wise sequence alignment between the two halves of the PepT$_{So}$ and PepT$_{St}$ sequences were constructed by superposing the structural repeats of each protein onto one another using the structure alignment program SKA (Petrey et al., 2003). Hence, repeat unit A of PepT$_{So}$ (residues 11-107) was superposed on repeat unit B (residues 108-201), and repeat unit C (residues 287-400) was superimposed on repeat unit D (residues 402-512) resulting in two alignments. The RMSD values between repeat units A & B and C & D are 2.1 and 4.0 Å, respectively. Likewise, repeat unit A of PepT$_{St}$ (residues 14-105) was superposed on repeat unit B (residues 106-201), and repeat unit C (residues 280-379) was superimposed on repeat unit D (residues 380-471) with RMSD values of 2.6 Å (A & B) and 3.5 Å (C & D). Each set of two sequence alignments was then spliced together to obtain a pair-wise alignment between the model sequence (either PepT$_{So}$ or PepT$_{St}$) and the corresponding template, which is the PepT$_{So}$ or PepT$_{St}$ sequence whose repeats have been rearranged so that they have the order BADC. The additional helices present in both protein, HA & HB, are not part of any repeat unit and so were omitted from all model building. Both preliminary alignments were then adjusted manually to remove gaps in the TM helices and the sequences of individual helices were shifted to improve the sequence conservation, resulting in the final alignments (Figure S10, S11). Even before these further adjustments to the preliminary alignment, the corresponding models are consistent with outward-facing conformations of PepT$_{So}$ and PepT$_{St}$.

1.3 Building the repeat-swapped models.

Using these sequence alignments the repeat-swapped models of PepT$_{So}$ and PepT$_{St}$ were constructed using Modeller 9.7 (Sali and Blundell, 1993). The inward-open PepT$_{So}$ (PDB:4UVM) and PepT$_{St}$ (PDB:4APS) crystal structures were used as templates (Solcan et al., 2012). Structural restraints were added to preserve the secondary structure of the models. Specifically, for PepT$_{So}$ we constrained the N-terminal tips of H2, H4, H5, H6, H7 and H11 and the C-terminal tips of H1, H2, H3 and H5 to be helical (for PepT$_{St}$ the equivalent helices were H5, H6, H9 & H10 and H1, H7, H8, H9, H10 and H11, respectively). In addition, the distance between the guanidine carbon and the side chain nitrogen of the charged pair R25 and K127 (R26 and K126 in PepT$_{St}$) was constrained to be 4.5 $\pm$ 0.5 Å. This prevented the arginine side chain from pointing into the hydrophobic lipid core. One thousand models were created of each protein and the models were then ordered according to the Modeller (DOPE) score.

The 100 PepT$_{So}$ and PepT$_{St}$ models with the lowest scores were selected for further refinement. The energy of the PepT$_{So}$ models was minimised prior to analysis and hydrogens were added using GROMACS (version 4.6) (Pronk et al., 2013). The energy minimisation was performed in three stages, each with 250 steps of steepest decent minimization, followed by 250 steps of conjugated gradient minimization. In the first stage, only the hydrogen atoms were allowed to move. In the second stage, only the hydrogen and side chain atoms were allowed to move. In the final stage, no constraints were applied. The minimization was performed using the CHARMM22 force field (MacKerell et al., 1998). To account for the fact that the central cavity is filled with water, a high dielectric constant ($\varepsilon = 80$) was used for the calculation of electrostatic interactions. The same procedure was repeated for the PepT$_{St}$ models, except CHARMM (version 3.4) (Brooks et al., 1983) was used to minimise the energy and the positions of hydrogen atoms were predicted using REDUCE (version 2.21) (Word et al., 1999). The two structures with the lowest Modeller (DOPE) scores were selected to be the representative, one for each protein. Both models had reasonable quality according to Procheck (Laskowski et al., 1993), with the PepT$_{So}$ model having only three and four residues (two and three for PepT$_{St}$) occupying generously allowed or disallowed regions of the Ramachandran plot, respectively.

1.4 Protein purification and crystallization.

Wild-type and mutant PepT$_{So}$ were purified to homogeneity as described previously (Newstead et al., 2011).

The protein-laden mesophase was prepared by homogenizing 7.8 MAG and 10 mg/ml protein solution in a 1:1 ratio by weight using a dual syringe mixing device at 20 °C (Caffrey and Cherezov, 2009). Crystallisation trials were carried out at 20

°C in 96-well glass sandwich plates with 50 nL mesophase and 0.8 mL precipitant solution using an *in meso* robot (Cherezov et al., 2004). Crystallisation solutions consisted of 20-23 %(v/v) PEG 400, 0.1 M sodium acetate pH 4.5-4.8 and 0.08-0.12 M sodium citrate. 3D bipyramidal crystals grew to a maximum size of 60 x 30 x 30 μm^3 in 1 to 5 days. Wells were opened using a tungsten-carbide glasscutter and the crystals were harvested using 30-50 μm micromounts (MiTeGen) (Li et al., 2012). Crystals were cryo-cooled directly in liquid nitrogen.

1.5 Data collection and processing.

X-ray diffraction data was collected on the I24 beamline at the Diamond Light Source, Oxford, UK. Data was acquired using a 10 μm microfocus beam. Oscillation data was measured in 0.2° frames with 0.2 s exposures. All data were initially reduced using XDS (Kabsch, 2010), XSCALE and AIMLESS (Evans, 2011; Winter et al., 2013) (Table S1).

1.6 Structure solution and refinement

Molecular replacement search models were prepared from the inward-occluded $PepT_{So}$ model (PDB ID: 2XUT) pruned of all side chains and non-protein atoms using Chainsaw (Collaborative Computational Project, 1994). Initial phases were obtained by MR using Phaser (McCoy et al., 2007). Iterative rounds of structure refinement were performed in PHENIX (Adams et al., 2010). The structural model was revised in real space with the program COOT (Emsley et al., 2010) using sigma-A weighted $2F_o$-F_c and mF_o-DF_c electron density maps. The geometric quality of the model was assessed with MolProbity (Chen et al., 2010). Lipid molecules and waters were assigned based on sigma-A weighted $2F_o$-F_c and F_o-F_c electron density maps contoured at 1σ using standard geometrical and chemical restraints.

1.7 Transport assay

Both $PepT_{So}$ and $PepT_{St}$ were reconstituted into *Escherichia coli* total lipids with egg PC liposomes and assayed using a proton-driven system as previously described (Solcan et al., 2012).

1.8 Preparation of $PepT_{So}$ double-cysteine mutants.

E. coli C43 (DE3) cells overexpressing the $PepT_{So}$-pWaldo-GFPe construct (Drew et al., 2006) were grown in TB at 37°C and expression was induced with 0.2mM IPTG at OD_{600}=0.6. The temperature was dropped to 25°C overnight. Cells were harvested, resuspended in 1x PBS and lysed in a cell disruptor at 30Kpsi. Cell lysates were centrifuged at 25,000g for 30min, and cell membranes were separated by ultracentrifugation at 130,000g for 2h at 4°C. Membranes were resuspended in 1x PBS and flash-frozen in liquid nitrogen for storage. Protein purification was carried out as described previously (Newstead et al., 2011).

1.9 Spin-labeling protocol.

The $PepT_{So}$ double-cysteine mutants were reduced for one hour at room temperature in 10 mM DTT in buffer (20 mM Tris, pH 7.5, 150 mM NaCl, 0.03% DDM, 30% glycerol). The solution was diluted 10X and salt-exchanged to remove excess DTT (5 ml HiTrapTM, GE healthcare). MTSL dissolved in DMSO (10mg/ml) was added at a 10X molar ratio to each labeling site, and incubated with the sample for three hours at room temperature. The spin label was removed as before, and the sample concentrated to 200-300 μM using a vivapsin concentrator with a 50 kDa molecular weight cut-off (Sartorius Stedium). For pulsed experiments, samples were loaded into 3 mm quartz tubes and flash frozen in liquid nitrogen to avoid water crystallization.

1.10 Pulsed DEER spectroscopy.

All measurements were carried out on a Bruker Elexsys 680 at X-band (~9.5 GHz) between 50 and 80 K using an overcoupled (Q ≈ 100) 3 mm ER4118X-MS3 resonator. The four-pulse (4p) DEER experiment with the sequence (Pannier et al., 2000)

$$\pi/2(\nu_{obs}) - \tau_1 - \pi(\nu_{obs}) - t' - \pi(\nu_{pump}) - (\tau_1 + \tau_2 - t') - \pi(\nu_{obs}) - \tau_2 - \text{echo}$$

and the three-pulse (3p) DEER with the sequence (Milov et al., 1981)

$$\pi/2(\nu_{obs}) - t - \pi(\nu_{pump}) - (\tau_1 - t) - \pi(\nu_{obs}) - \tau_1 - \text{echo}$$

were both used. For both the 3p and 4p DEER experiments, the observer π and $\pi/2$ pulses were 32 ns, and the pump π pulse was 12 ns. A two-step phase cycle ($+x$ $-x$) was applied to the first observer pulse, and t was incremented in 8 ns

steps. The observer frequency coincided with the maximum of the nitroxide spectrum and the center of the microwave mode of the resonator, while the pump pulse was 65 MHz upfield. Accumulation times were between approximately four and sixteen hours, and the repetition rate adjusted to avoid signal saturation. τ was varied between 2000-3400 ns in the 3p DEER experiment, and between 800 and 2500 ns in the 4p DEER experiment to maximize signal to noise. For samples where both 3p and 4p DEER were collected, the DEER datasets were computationally combined such that one has to access both the zero time in the 4p DEER data, as well as longer evolution times due to the increased sensitivity in the 3p DEER experiment (Lovett et al., 2012). Data were processed and analyzed using DeerAnalysis2011 (Jeschke et al., 2006).

1.11 Least squares fit method for combining 3p and 4p DEER.

To combine the two datasets, the 3p and 4p DEER raw data were phased in DEERAnalysis 2011 to correct for any experimental data collected in the imaginary channel. Next, the zero times for the 3p and 4p datasets were defined. Distorted data points were removed from the end of the 4p DEER dataset, and the beginning of the 3p DEER dataset. Finally, each dataset was background-corrected simultaneously assuming a homogenous, spatially confined background,

$$B(t) = \exp(-kt^{(d/3)})$$

where k quantifies the concentration of the spins, and d is the background dimensions (typically two for membrane proteins in bilayers, and three when in detergent micelles). k is varied such that differences between the background decay of the two datasets are minimized to obtain the best fit between the two experimental datasets.

1.12 Determining the conformational state of MFS transporters.

The high resolution crystal structure of $\mathrm{PepT_{So}}$ was manually arranged so that the z-axis was parallel to the membrane normal and the origin of the coordinate system was located in the central cavity. HOLE (version 2.2) (Smart et al., 1996) was then run to find the maximum radius of a spherical probe that could be fitted at each value of z (Figure S4). This analysis was repeated for all other current structures of MFS transporters. All structures were fitted onto the structure of $\mathrm{PepT_{So}}$ using LOVOALIGN (Martínez et al., 2007). The minimum values of the probe radius in the two regions defined by the z values of the top or bottom two turns of the transmembrane helices was calculated, thereby identifying the constrictions that would create the periplasmic or cytoplasmic gates, respectively. Since the probe radius can vary rapidly with z, creating sharp peaks, we arbitrarily defined the probe radius for each gate as the average probe radius over a window 4 Å wide centred on the minimum value.

We determined which pairs of helices constituted the periplasmic and cytoplasmic gates by correlating the smallest distance between each pair of pairs and the gate probe radius defined above. Each gate was assumed to consist of two pairs of helices with the N- and C-terminal halves of the protein contributing one pair each. Due to the topology of MFS transporters, there are three and two helix pairs on the periplasmic and cytoplasic sides of each half of the protein, respectively, and hence there are nine and four possible combinations for the periplasmic and cytoplasmic gates. We wrote a python program, using the MDAnalysis module (Michaud-Agrawal et al., 2011), to calculate the minimum C_α-C_α distances between the pairs of helices. To ensure that the closest distance occurs on the periplasmic or cytoplasmic side of the protein, only the first (or last, as appropriate) ten residues were considered. The locations of the transmembrane helices were defined using STRIDE (Frishman and Argos, 1995).

1.13 Molecular dynamics simulations.

Molecular dynamics simulations of chain A from the experimental structure of apo $\mathrm{PepT_{So}}$ (PDB:2XUT) were carried out as described previously by Newstead et al. (2011). All amino acid side chains were set to their standard protonation states as there was no information about which side chains may become protonated during transport. Waters and neutralising ions were added creating a simulation unit cell containing 205 POPC lipids, 7 chloride ions and 13 711 water molecules making a total of 76 245 atoms. This was procedure was repeated for $\mathrm{PepT_{St}}$ (PDB:4APS) (Solcan et al., 2012) resulting in a simulation unit cell containing 250 POPC lipids, 6 chloride ions and 12 429 water molecules making a total of 78 033 atoms. Three independent molecular dynamics simulations were run for each protein, each 200 ns long, making a total of 1.2 μs. All coordinates were recorded every 10 ps.

1.14 Mapping MTSL spin labels onto structures and trajectories.

We used a program to fit MTSL rotamers onto protein structures and MD trajectories (Stelzl et al., 2014). This incorporates a published rotamer MTSL library (Polyhach et al., 2011) and is implemented in python using the MDAnalysis module (Michaud-Agrawal et al., 2011). The python code is available from the MDAnalysis website (http://code.google.com/p/mdanalysis/). All images were produced with VMD (Humphrey et al., 1996) using the bendix plugin (Dahl et al., 2012) and all graphs produced with gnuplot.

2 Supplemental Figures and Tables

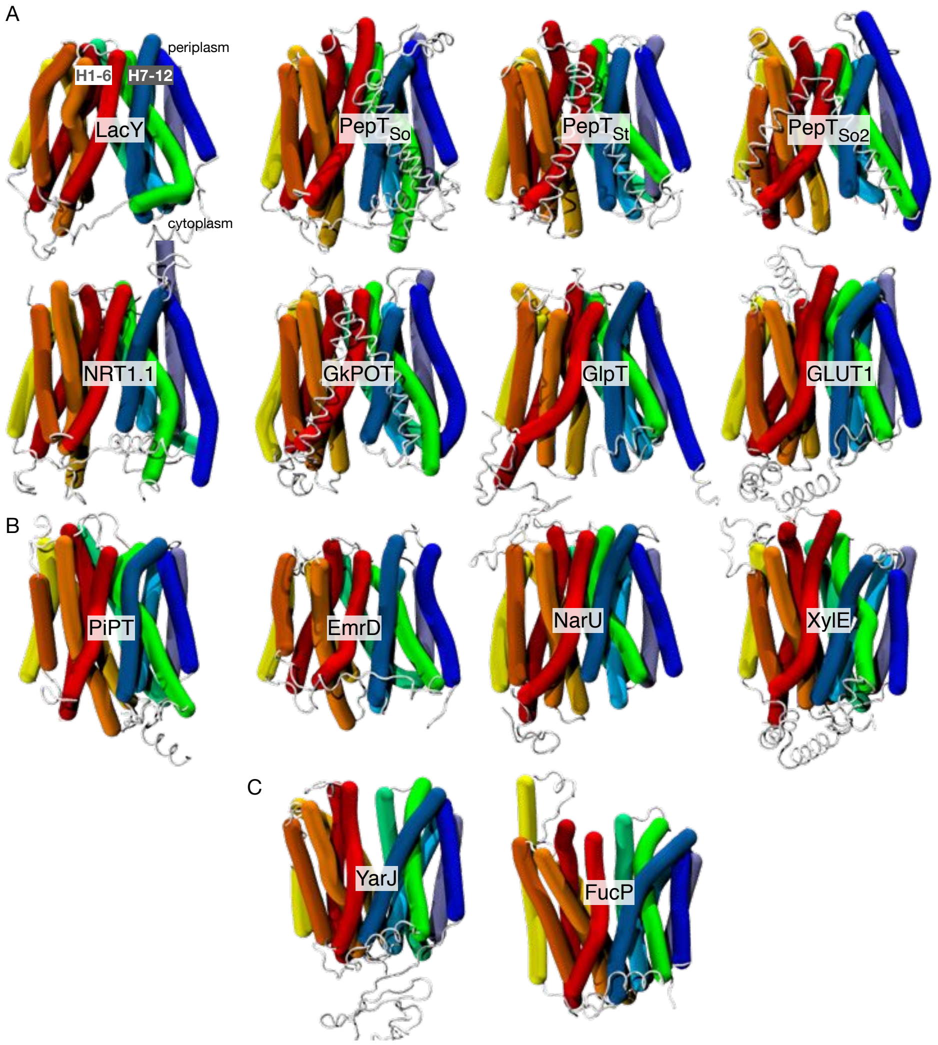

Figure S1: Related to Figure 1. Major Facilitator Superfamily transporters comprise two bundles of six helices. The central binding site is exposed to either side of the membrane (or neither, the occluded state). The known structures are pictured here ordered by conformation from (A) inward-facing (LacY (Abramson et al., 2003), PepT$_{So}$(Newstead et al., 2011), PepT$_{St}$(Lyons et al., 2014; Solcan et al., 2012), PepT$_{So2}$(Guettou et al., 2013), NRT1.1 (Parker and Newstead, 2014), GkPOT (Doki et al., 2013), GlpT (Huang et al., 2003), GLUT1 (Deng et al., 2014)) through (B) occluded (PiPT (Pedersen et al., 2013), EmrD (Yin et al., 2006), NarU (Yan et al., 2013), XylE (Quistgaard et al., 2013; Sun et al., 2012)) to (C) outward-facing (YarJ (Jiang et al., 2013) and FucP (Dang et al., 2010)). Although several proteins have been captured in more than one conformation we only show one example of each here for clarity. The colour scheme is in the same as used in Figure 1.

	RMSD between inward-open structure and …		RMSD between outward-open model and …	
	inward-facing conformations	outward-facing conformations	inward-facing conformations	outward-facing conformations
**$\mathbf{PepT_{So}}$ MD sims**				
6-TM helix bundles				
half 1	2.0 ± 0.1	2.2 ± 0.0	2.9 ± 0.1	2.8 ± 0.1
half 2	2.4 ± 0.0	2.8 ± 0.0	4.9 ± 0.1	4.7 ± 0.0
Inverted topology repeats				
unit 1	1.7 ± 0.1	1.9 ± 0.1	2.5 ± 0.1	2.5 ± 0.0
unit 2	1.9 ± 0.1	2.2 ± 0.1	2.4 ± 0.1	2.8 ± 0.0
unit 3	2.4 ± 0.1	2.8 ± 0.0	4.1 ± 0.1	4.0 ± 0.0
unit 4	1.7 ± 0.1	2.4 ± 0.1	4.1 ± 0.1	4.8 ± 0.1
Transmembrane helices				
helix 1	1.3 ± 0.1	1.8 ± 0.0	1.5 ± 0.1	1.6 ± 0.0
helix 2	1.9 ± 0.2	1.3 ± 0.0	3.1 ± 0.2	2.3 ± 0.1
helix 3	0.7 ± 0.0	0.5 ± 0.0	1.1 ± 0.0	0.9 ± 0.0
helix 4	1.0 ± 0.1	1.1 ± 0.1	1.5 ± 0.1	1.4 ± 0.0
helix 5	2.1 ± 0.1	1.6 ± 0.1	2.6 ± 0.1	2.8 ± 0.1
helix 6	0.7 ± 0.1	0.6 ± 0.1	1.0 ± 0.1	1.0 ± 0.0
helix 7	2.0 ± 0.1	2.6 ± 0.1	4.0 ± 0.1	3.3 ± 0.0
helix 8	2.2 ± 0.0	2.1 ± 0.1	2.4 ± 0.1	2.3 ± 0.0
helix 9	0.8 ± 0.1	0.9 ± 0.0	0.9 ± 0.1	1.2 ± 0.0
helix 10	1.3 ± 0.1	1.7 ± 0.0	3.7 ± 0.1	3.8 ± 0.0
helix 11	1.1 ± 0.1	0.8 ± 0.0	1.3 ± 0.1	1.4 ± 0.0
helix 12	0.7 ± 0.1	0.6 ± 0.0	0.9 ± 0.1	0.8 ± 0.1
**$\mathbf{PepT_{St}}$ MD sims**				
6-TM helix bundles				
half 1	2.0 ± 0.1	2.2 ± 0.0	2.9 ± 0.1	2.8 ± 0.1
half 2	2.4 ± 0.0	2.8 ± 0.0	4.9 ± 0.1	4.7 ± 0.0
Inverted topology repeats				
repeat unit 1	1.7 ± 0.1	1.9 ± 0.1	2.5 ± 0.1	2.5 ± 0.0
repeat unit 2	1.9 ± 0.1	2.2 ± 0.1	2.4 ± 0.1	2.8 ± 0.0
repeat unit 3	2.4 ± 0.1	2.8 ± 0.0	4.1 ± 0.1	4.0 ± 0.0
repeat unit 4	1.7 ± 0.1	2.4 ± 0.1	4.1 ± 0.1	4.8 ± 0.1
Transmembrane helices				
helix 1	1.3 ± 0.1	1.8 ± 0.0	1.5 ± 0.1	1.6 ± 0.0
helix 2	1.9 ± 0.2	1.3 ± 0.0	3.1 ± 0.2	2.3 ± 0.1
helix 3	0.7 ± 0.0	0.5 ± 0.0	1.1 ± 0.0	0.9 ± 0.0
helix 4	1.0 ± 0.1	1.1 ± 0.1	1.5 ± 0.1	1.4 ± 0.0
helix 5	2.1 ± 0.1	1.6 ± 0.1	2.6 ± 0.1	2.8 ± 0.1
helix 6	0.7 ± 0.1	0.6 ± 0.1	1.0 ± 0.1	1.0 ± 0.0
helix 7	2.0 ± 0.1	2.6 ± 0.1	4.0 ± 0.1	3.3 ± 0.0
helix 8	2.2 ± 0.0	2.1 ± 0.1	2.4 ± 0.1	2.3 ± 0.0
helix 9	0.8 ± 0.1	0.9 ± 0.0	0.9 ± 0.1	1.2 ± 0.0
helix 10	1.3 ± 0.1	1.7 ± 0.0	3.7 ± 0.1	3.8 ± 0.0
helix 11	1.1 ± 0.1	0.8 ± 0.0	1.3 ± 0.1	1.4 ± 0.0
helix 12	0.7 ± 0.1	0.6 ± 0.0	0.9 ± 0.1	0.8 ± 0.1

Table S1: Relates to Figure 6. The C-terminal half of $PepT_{St}$ is more dynamic than the N-terminal half. The inward-open and outward-open structures in the ensemble of structures generated by the 600 ns molecular dynamics of both proteins were identified. Components of each structure were aligned back onto both the relevant inward-open crystal structure (or outward-open repeat swapped model) and the C_α RMSD calculated (in Å).

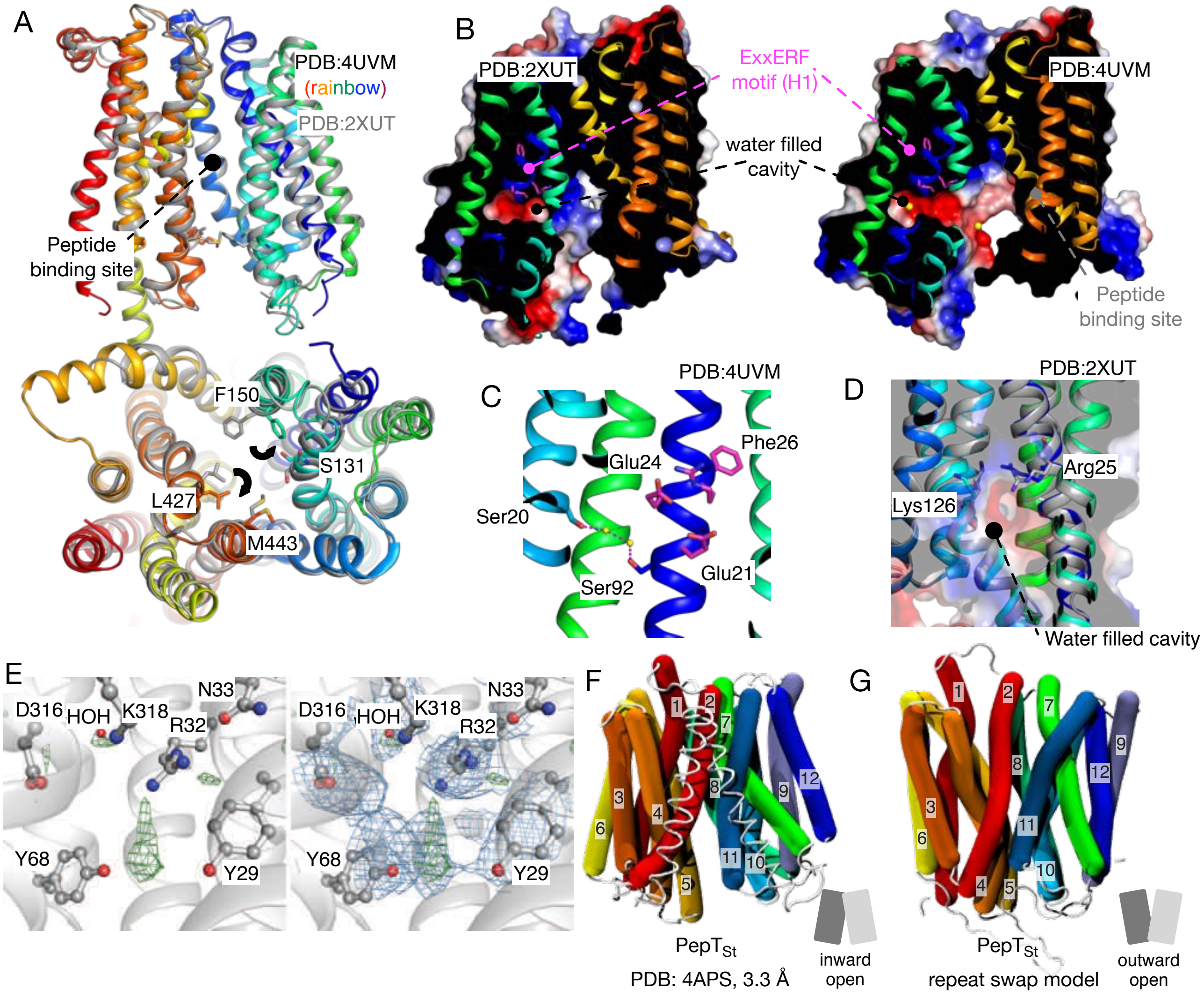

Figure S2: Related to Figure 2. (A) An overlay of the new and existing structures of PepT$_{So}$ showing that although the structures are similar there are key differences in the positions of the residues that make up the cytoplasmic 'thin' gate. (B) The previous occluded structure of PepT$_{So}$ (2XUT) contained a hydrophilic cavity adjacent to the E21xxERF motif on helix H1. In the new inward open structure (4UVM) this cavity is also observed. (C) Interestingly two well coordinated water molecules sit at the far end of the cavity, making hydrogen bonds to Ser20 (H1) and Ser92 (H3). Previously we showed that the ExxERF motif on helix H1 plays an important role in coupling peptide transport to the inward movement of protons (Solcan et al., 2012). The inward open structure of PepT$_{So}$ now provides a mechanism by which Glu21 and/or Glu24 could exchange protons with the bulk solvent. (D) A structural overlay with the occluded structure (2XUT, grey) and inward open structure (4UVM, coloured helices) reveals that in the inward-occluded 2XUT structure the tunnel is closed through the close positioning of Arg25 (H1) and Lys126 (H4). The functional significance of this with respect to the transport mechanism however is currently unclear, but may suggest a dynamic mechanism allowing water access to this region of the transporter. (E) The new structure of PepT$_{So}$ contains an unknown ligand sitting in the peptide binding site. An unidentified ligand is observed sitting in the central peptide binding site, as revealed by the positive difference density map (F_o-F_c, green/red, contoured at $\pm 3.5\ \sigma$). This observation suggests that the current structure (PDB: 4UVM) represents a a possible ligand bound inward open state, whereas the previously reported structure (PDB: 2XUT) represents an occluded inward open state. The second panel shows the same view but with the $2F_o$-F_c electron density map, contoured at 1 σ. (F) The inward-open structure of PepT$_{St}$ with the helices rendered using curved cylinders (Dahl et al., 2012) to illustrate their intrinsic kinks and bends (Solcan et al., 2012). The two additional helices found in the bacterial proton oligopeptide transporters, HA and HB, are coloured light grey. (G) An outward-open model of PepT$_{St}$, built using the repeat-swapping method. The transmembrane helices are coloured according to the same scheme throughout. Equivalent images to panels F and G for PepT$_{So}$ can be found in Figure 2.

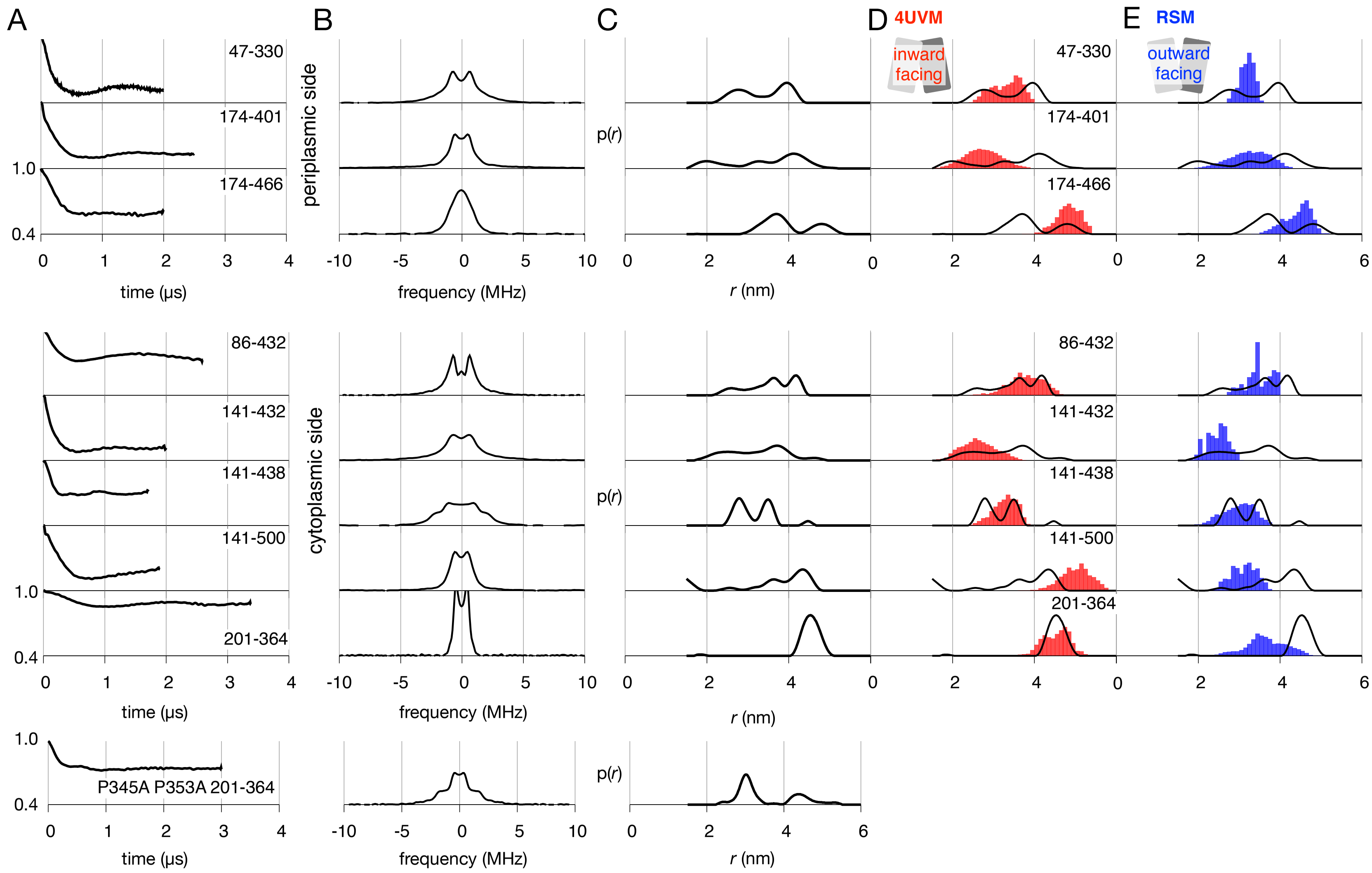

Figure S3: Related to Figure 3. The outward-facing model of $PepT_{So}$ can be validated against the spin-spin distance distributions inferred from the DEER experiments. (A) Time domain data for the three periplasmic and five cytoplasmic distances studied by DEER, including the double proline mutant of the 201-364 distance. (B) Pake patterns obtained after Fourier transformation of the time domain data. (C) Measured DEER spin-spin distance distributions, following Tikhonov regularization. (D) A comparison between the predicted spin-spin distance distribution (in red) for the inward-open crystal structure (PDB:4UVM) and the measured DEER distance distributions. A library of MTSL rotamers was mapped onto each position and then any that clashed with the protein were discarded. (E) A comparison between the predicted spin-spin distance distributions (in blue) for the outward-open repeat-swapped model (RSM) and the DEER distance distributions.

Figure S4: Related to Figure 4. The percolation surfaces and pore profiles for all known structures of MFS transport proteins. This was calculated using HOLE (Smart et al., 1996) as described in the Experimental Procedures. The surface is coloured according to the maximum radius of the spherical probe; less than 1.15 Å is coloured red, greater than 2.30 Å yellow and in between, orange. The pore profile (the variation in the maximum radius of a spherical probe as a function of *z*) can be used to identify constrictions. The maximum radius of a probe that can pass any constriction is estimated as the average of the probe radius over a window 4 Å wide centred on the constriction (i.e. the minimum value). The periplasmic and cytoplasmic gate regions in the pore profile are coloured light green and cyan and the 4 Å windows coloured dark green and dark blue, respectively.

(a) The percolation surfaces through 16 structures of members of the MFS. The PDB code of each structure is given in parentheses.

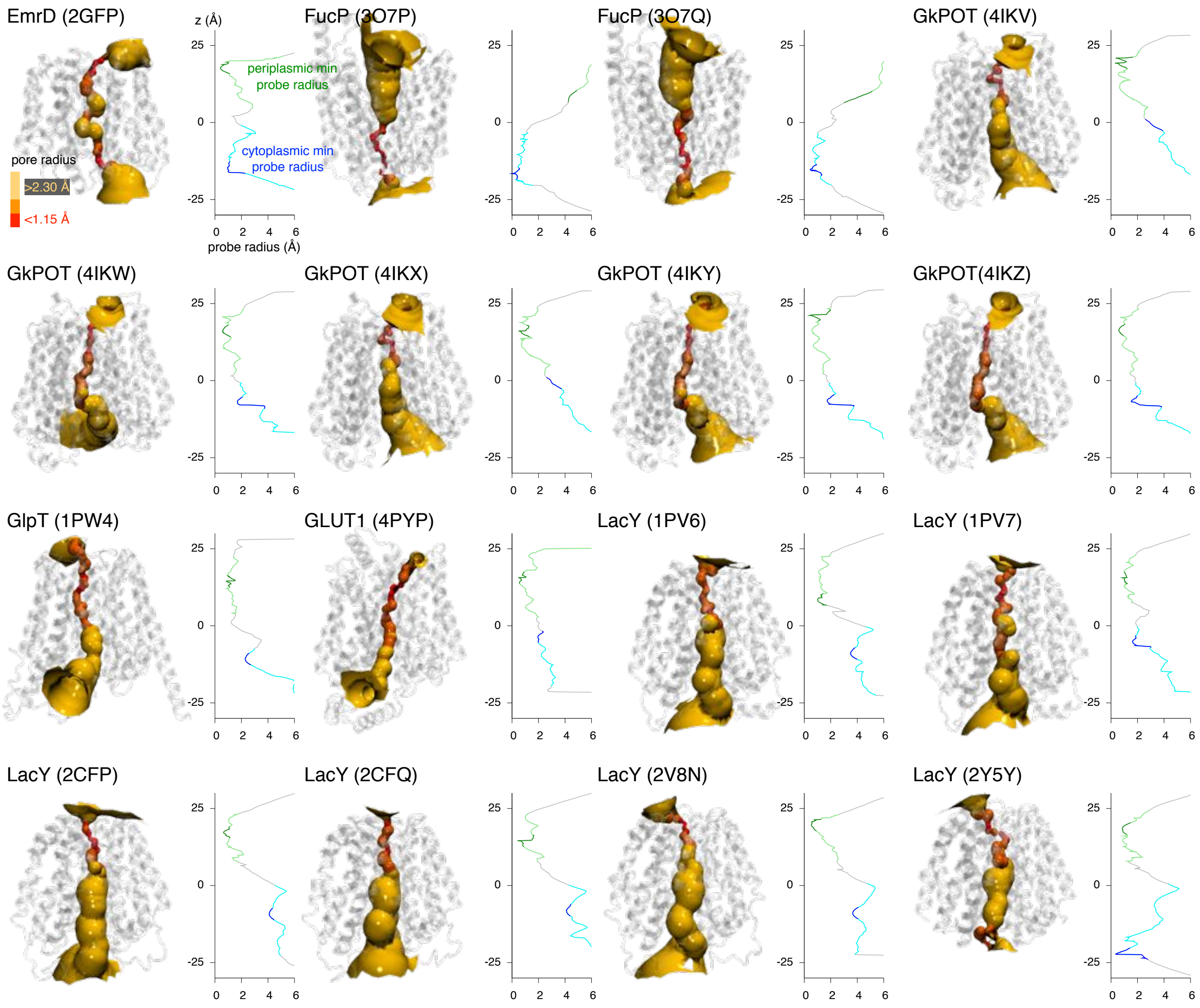

(b) The percolation surfaces through the remaining 16 structures of members of the MFS. The PDB code of each structure is given in parentheses.

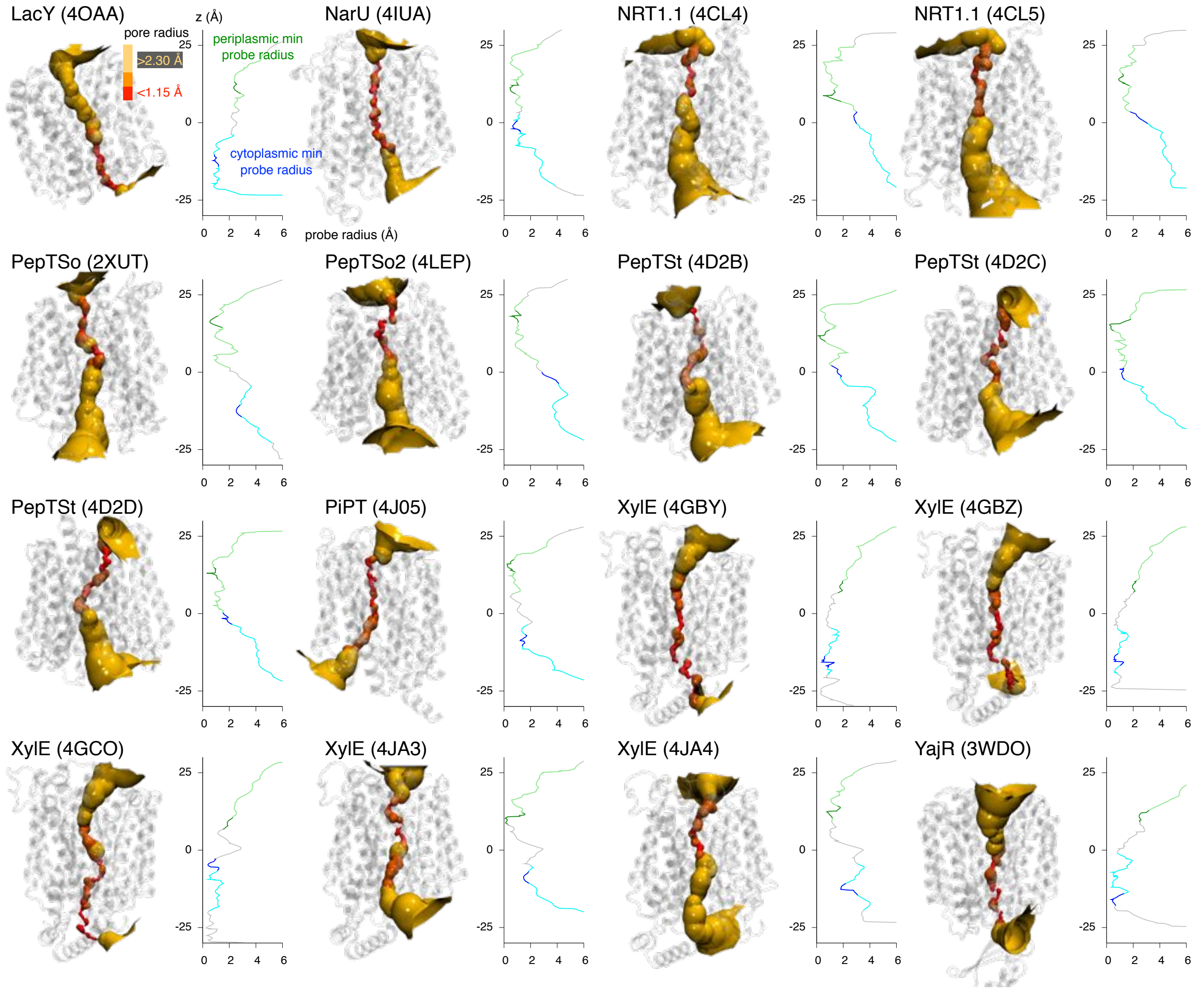

(c) The percolation surfaces through the structures of $PepT_{St}$ and its repeat-swapped model

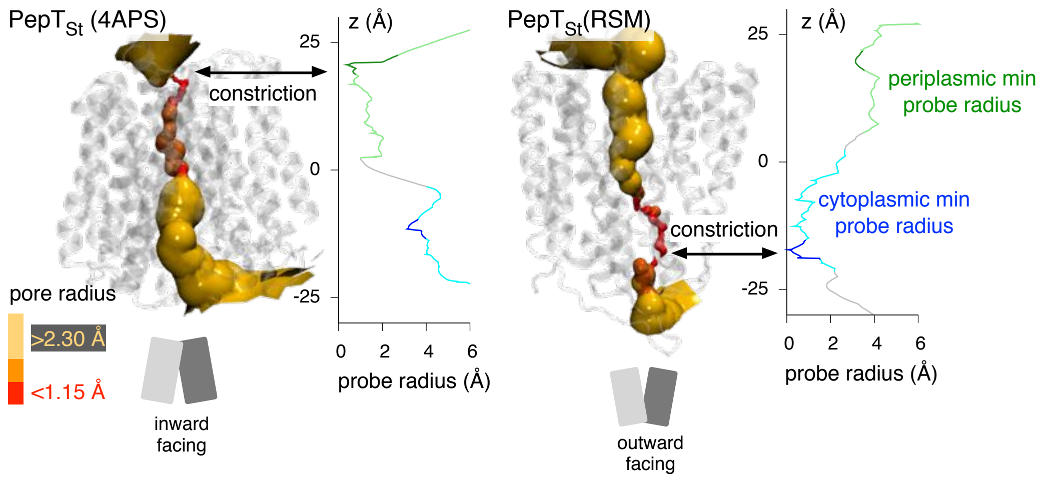

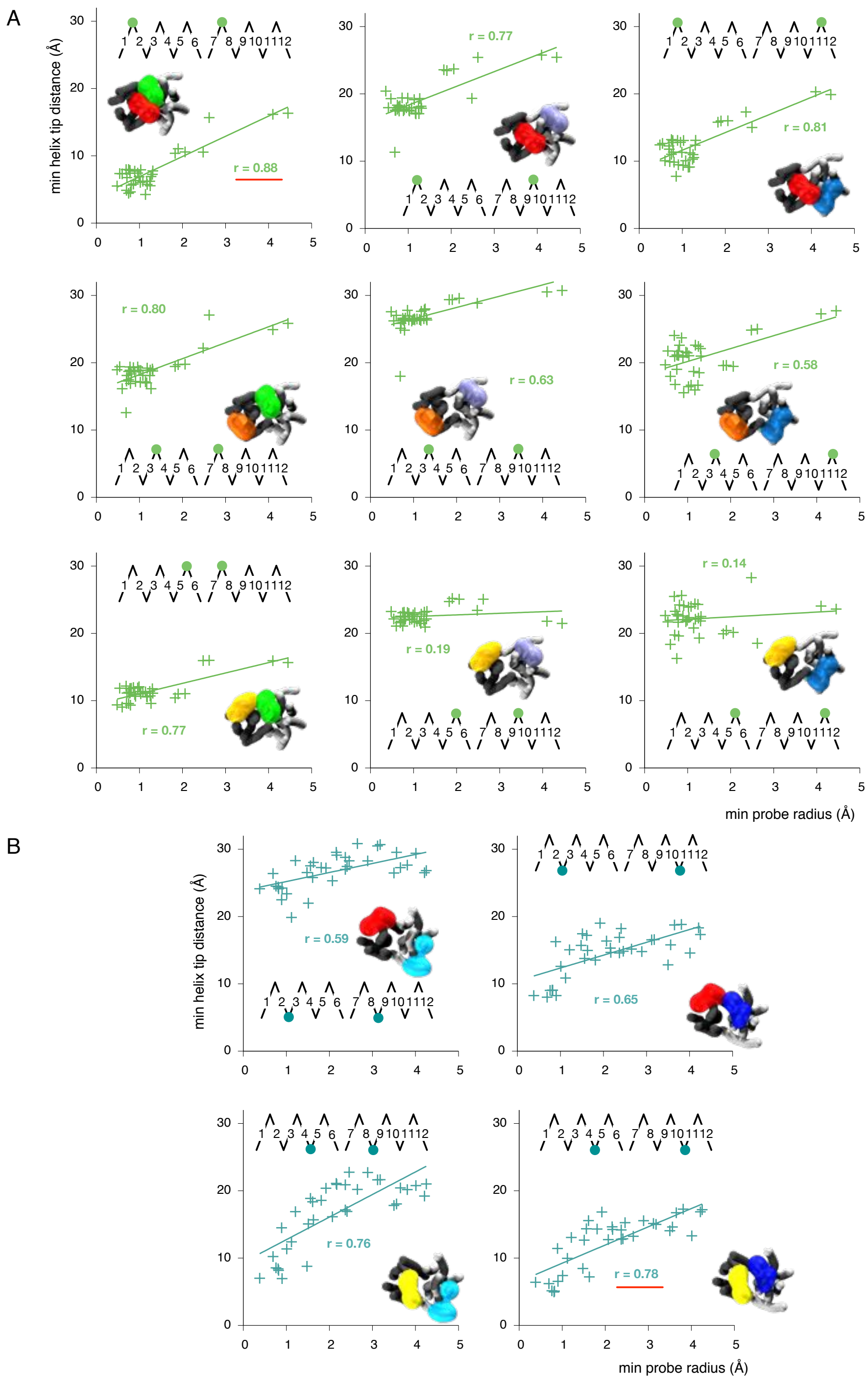

Figure S5: Related to Figure 5. MFS transporters are made up of two bundles of six helices with the N-terminus of the first helix starting in the cytoplasm. In each half of the transporter there are therefore three helix pairs that could contribute to the periplasmic gate (H1 & H2, H3 & H4, H5 & H6 and H7 & H8, H9 & H10, H11 & H12) and two helix pairs that could contribute to the cytoplasmic gate (H2 & H3, H4 & H5 and H8 & H9, H10 & H11) – see Figure 5A. If we assume that each gate is made up of one helix pair from the first six helices and one helix pair from the second six helices, then there are (A) nine possible combinations for the periplasmic gate and (B) four for the cytoplasmic gate. To determine which helices form the gates, we have plotted the minimum C_α-C_α distance as described in the Methods against the (cytoplasmic or periplasmic) minimum probe radius as determined by HOLE (see Figure 4, S4). The Pearson correlation coefficient is calculated for each set of data: this indicates that the minimum helix tip distance between H1 & H2 and H7 & H8 correlates best with the state of the periplasmic gate (R=0.88) and the minimum helix tip distance between H4 & H5 and H10 & H11 correlates best with the state of the cytoplasmic gate (R=0.78. Twenty nine different MFS structures were used.

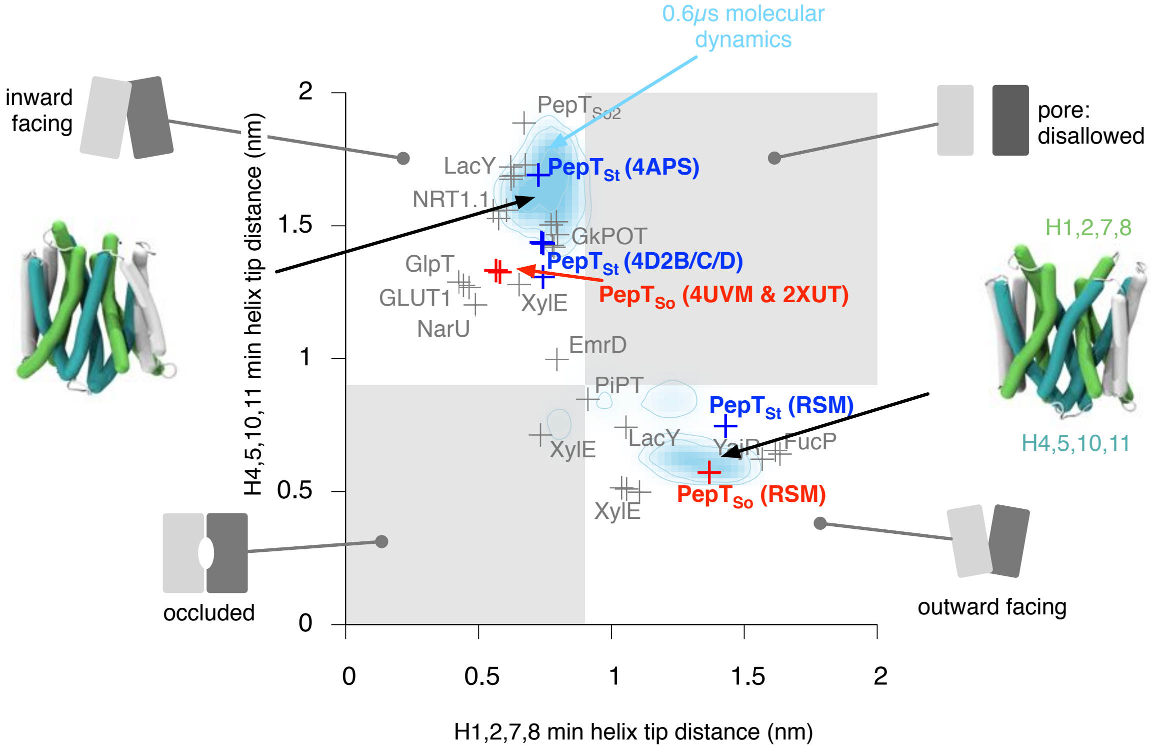

Figure S6: Related to Figure 6. During the molecular dynamics simulations PepT$_{St}$ explores inward-inward and some partially outward-facing conformations, as defined by the minimum distance between the C$_\alpha$atoms of the relevant pairs of helix tips. The density of states explored during the simulations are plotted in blue and two representative inward-facing and outward-facing structures are shown. The coordinates of known MFS structures are plotted to provide some context and the different quadrants of the coordinate space are labelled. The coordinates of the PepT$_{So}$ and PepT$_{St}$ crystal structures and repeat-swapped models (RSM) are labelled in red and blue, respectively. The results from the PepT$_{So}$ simulations can be found in Figure 6.

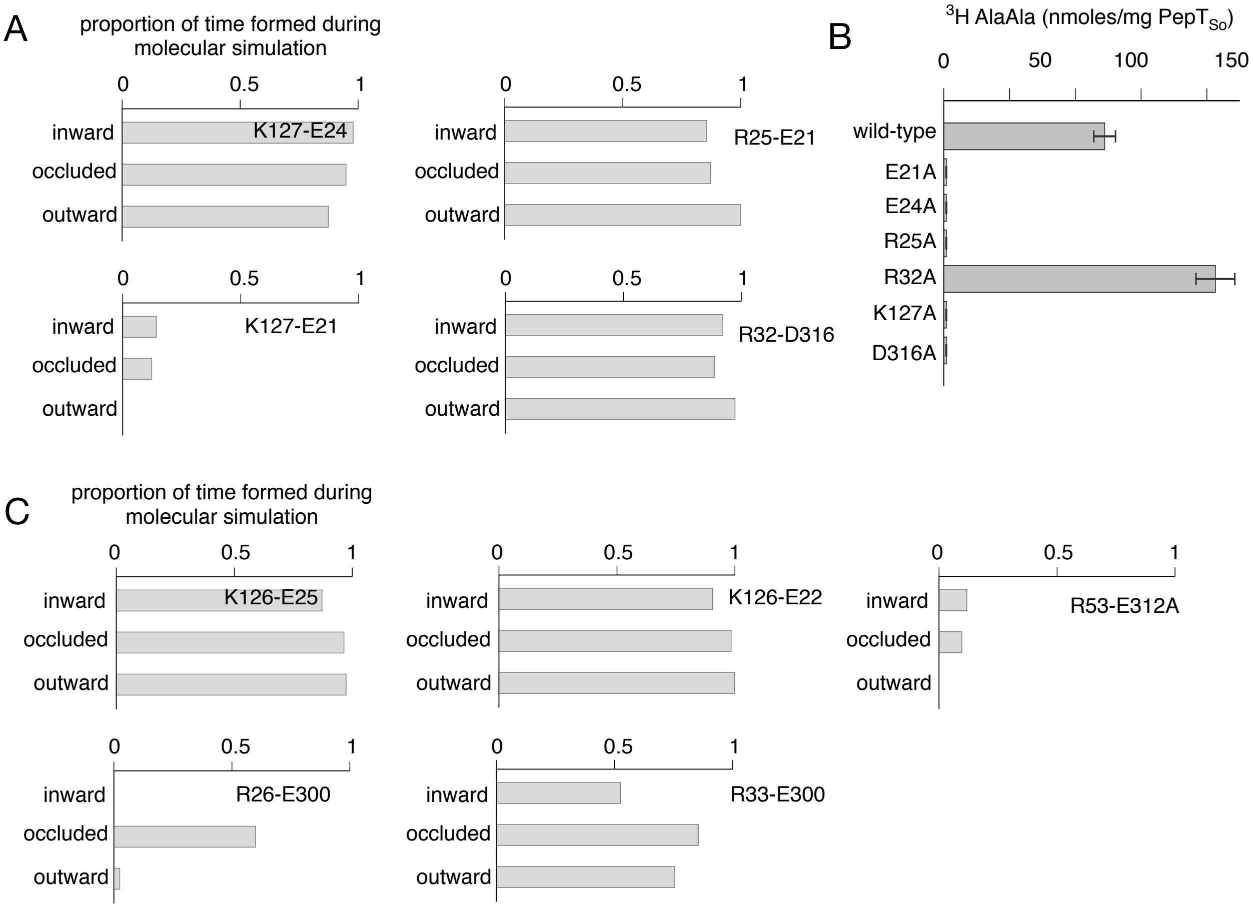

Figure S7: Related to Figure 7. The molecular dynamics simulations predict several salt bridges in the different conformations of PepT$_{So}$ and PepT$_{St}$. (A) Four salt bridges additional to those given in Figure 7 were identified in PepT$_{So}$. The propensity of three of these (K127-E24, R25-E21 and R32-D316) was found to be uniformly high across all the conformations sampled and K127-E21 was predicted to form only occasionally in inward and occluded conformations. Two of these interactions (K127-E24 and K127-E21) join helices (H1 & H4) involved in the periplasmic and cytoplasmic gates. Asp316 forms part of the GDQF signature motif on H4, except in PepT$_{St}$ which does not possess this motif. Several of the other residues belong to the conserved ExxERFxYY motif on H1, which has been identified as playing an important role in both proton-coupling and peptide recognition in PepT$_{St}$ (Solcan et al., 2012). Apart from the exception mentioned above, all of the residues are conserved from PepT$_{So}$ to mammalian PepT1 (Figure S8). These observations are consistent with (but do not prove) the hypothesis that these salt bridges are essential for the function of PepT$_{So}$. (B) Mutating any of the residues involved in these salt bridges abolishes transport, with the exception of R32A which has increased active transport compared to wild-type. (C) Five salt bridges were identified in the simulations of PepT$_{St}$. Two of these (R33-E300 and R53-E312) have been previously suggested to stabilise inward-facing conformations (Solcan et al., 2012). We find that this salt bridge only occurs in ~ 10 % of inward and occluded conformations and hence is unlikely to be critical, consistent with the transport data. The equivalent interaction (R52-D328) was detected in the simulations of PepT$_{So}$ – it too, only occurred in a minority of inward and occluded conformations and mutation to alanine also reduced but did not abolish active transport. Of the remaining three salt bridges, two (E22-K126 and E25-K126) are equivalent to those seen in PepT$_{So}$. This leaves R26-E300 which is predicted to occur in occluded conformations of PepT$_{St}$. Mutating any of E22, E25, R26, R33, K126 or E300 has been previously shown to abolish or significantly reduce transport (Solcan et al., 2012), again consistent with (but not proving) the hypothesis that these salt bridges stabilise PepT$_{St}$.

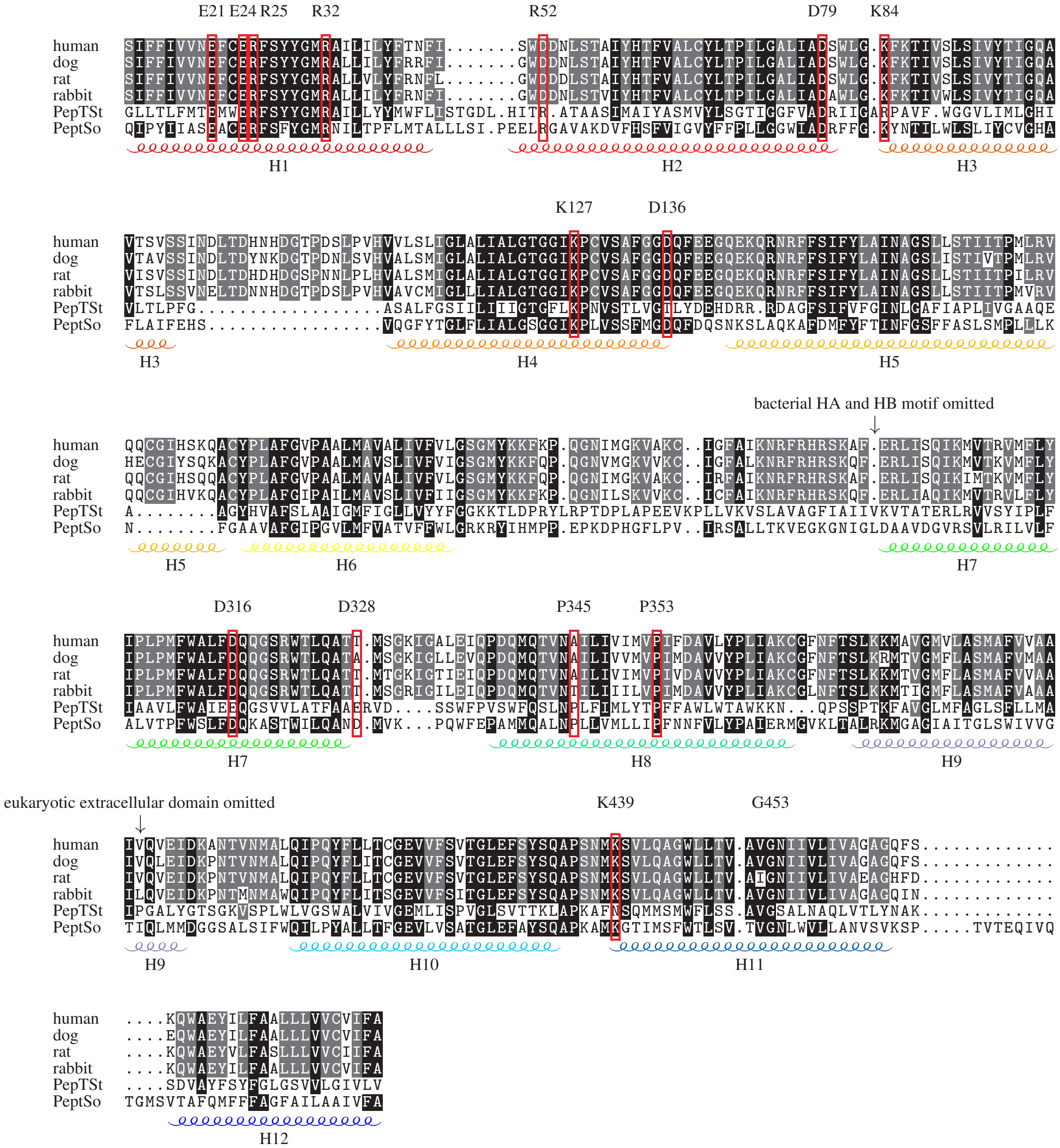

Figure S8: Refers to Figure 8. $PepT_{So}$ and $PepT_{St}$ are homologous to human PepT1. A multiple sequence alignment of PepT1 from four mammalian species and two bacterial peptide transporters, $PepT_{So}$ Newstead et al. (2011) and $PepT_{St}$ Solcan et al. (2012). Conserved residues are highlighted and key features, derived from the structure of $PepT_{So}$ are labeled Newstead et al. (2011). These include all twelve transmembrane helices and several residues (in red) referred to in the main body of the manuscript. All residue numbering is based on $PepT_{So}$, as are the definitions of the transmembrane helices. For clarity two regions are not shown: these are the extracellular domain (ECD) in PepT1 that has no equivalent in the bacterial peptide transporters and the additional two helices, HA and HB, in the bacterial peptide transporters that have no equivalent in the mammalian peptide transporters.

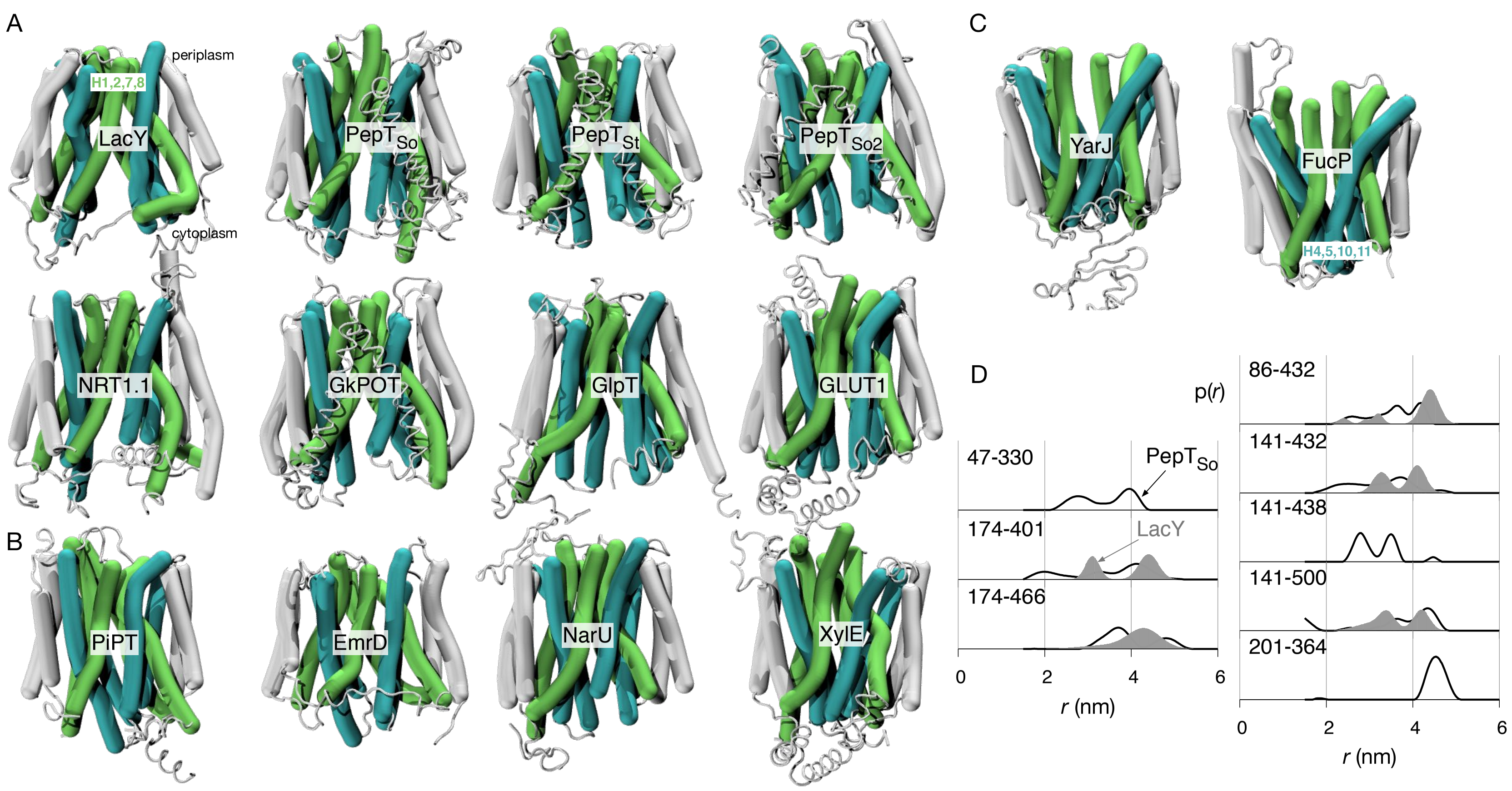

Figure S9: Related to Figure 9. Images of the current structures of MFS transporters coloured with the helices making up the periplasmic and cytoplasmic gates highlighted. Helices H1, H2, H7 & H8 are coloured green, whilst helices H7, H8, H10 & H11 are coloured cyan. The known structures are pictured here ordered by conformation from (A) inward-facing (LacY (Abramson et al., 2003), PepT$_{So}$(Newstead et al., 2011), PepT$_{St}$(Lyons et al., 2014; Solcan et al., 2012), PepT$_{So2}$(Guettou et al., 2013), NRT1.1 (Parker and Newstead, 2014), GkPOT (Doki et al., 2013), GlpT (Huang et al., 2003), GLUT1 (Deng et al., 2014)) through (B) occluded (PiPT (Pedersen et al., 2013), EmrD (Yin et al., 2006), NarU (Yan et al., 2013), XylE (Quistgaard et al., 2013; Sun et al., 2012)) to (C) outward-facing (YarJ (Jiang et al., 2013) and FucP (Dang et al., 2010)). Although several proteins have been captured in more than one conformation we only show one example of each here for clarity.(D) Five of the eight distances studied with DEER are structurally equivalent to distances studied in LacY, the canonical member of the MFS (**?**). The LacY DEER study used the same spin label and a non-binding sugar, 4-nitrophenyl-α-D-glucopyranoside (NPGlc), was added. The DEER distance distributions for LacY (shown as a grey shaded area) are very different to those of PepT$_{So}$, with not only the positions of peaks, but also the number of peaks varying between the two transporters.

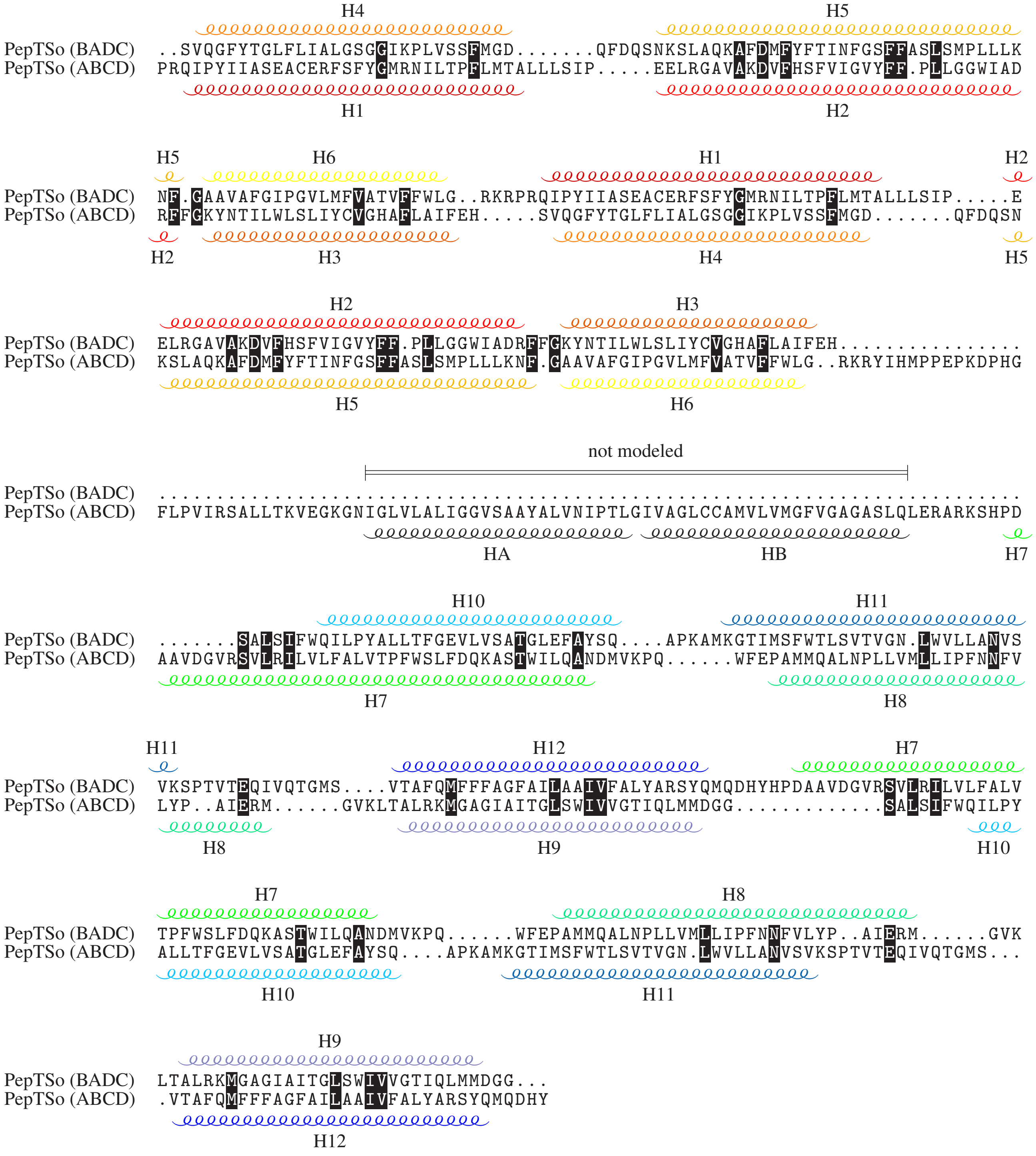

Figure S10: Related to the Experimental Procedures. Wildtype $PepT_{So}$ aligned onto $PepT_{So}$ with its alternating repeats swapped (ABCD to BACD). This alignment was used to construct the outward-open model of $PepT_{So}$. The helices are coloured as in Fig. 1.

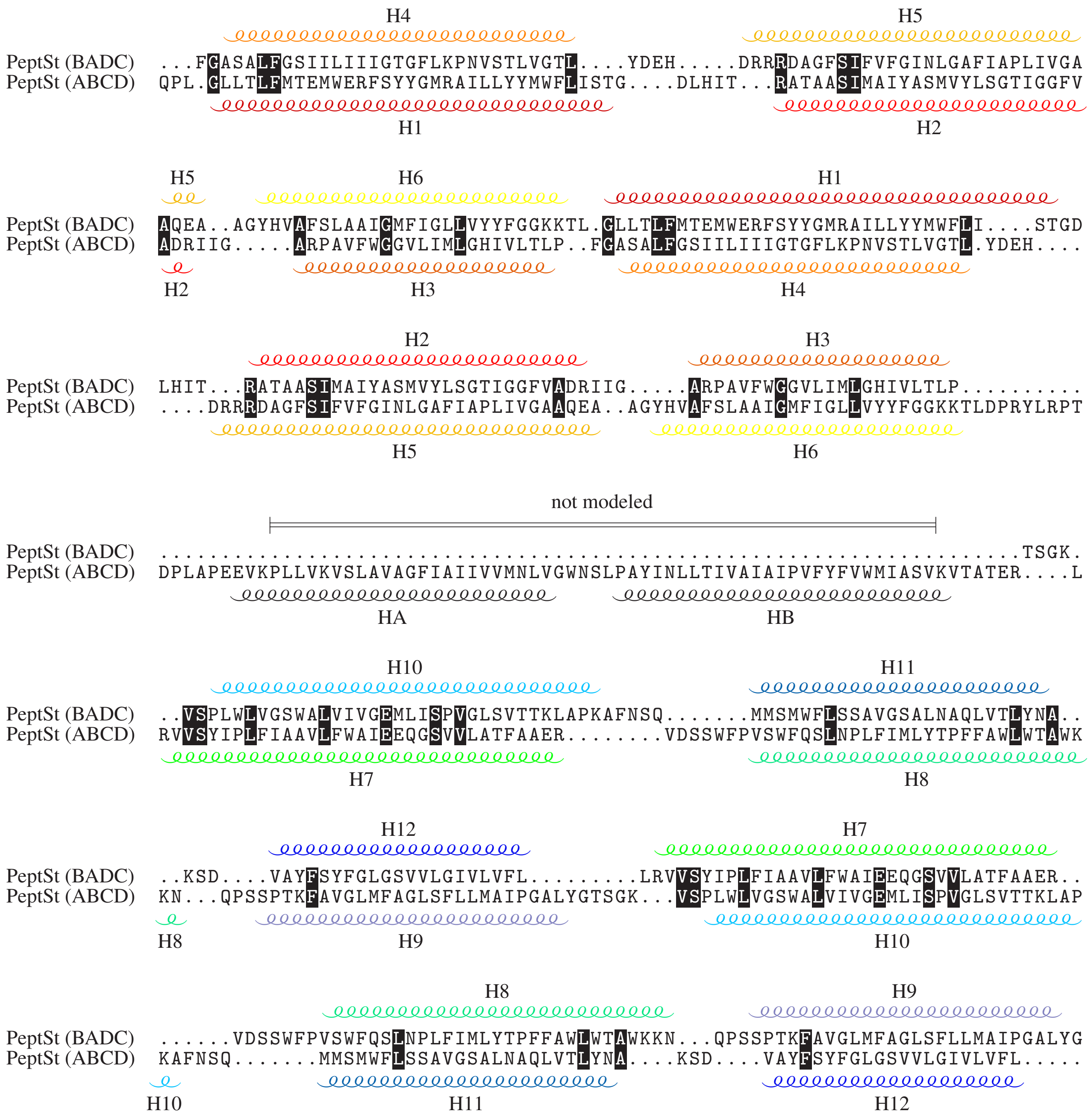

Figure S11: Related to the Experimental Procedures. Wildtype $PepT_{St}$ aligned onto $PepT_{St}$ with its alternating repeats swapped (ABCD to BACD). This alignment was used to construct the outward-open model of $PepT_{St}$. The helices are coloured as in Fig. 1.

References

Abramson, J., Smirnova, I., Kasho, V., Verner, G., Kaback, H.R., and Iwata, S. (2003). Structure and mechanism of the lactose permease of Escherichia coli. Science *301*, 610–5.

Adams, P.D., Afonine, P.V., Bunkóczi, G., Chen, V.B., Davis, I.W., Echols, N., Headd, J.J., Hung, L.W., Kapral, G.J., Grosse-Kunstleve, R.W., et al. (2010). PHENIX: a comprehensive Python-based system for macromolecular structure solution. Acta Cryst D *66*, 213–21.

Brooks, B.R., Bruccoleri, R.E., Olafson, B.D., States, D.J., Swaminathan, S., and Karplus, M. (1983). CHARMM: A program for macromolecular energy, minimization, and dynamics calculations. J Comput Chem *4*, 187–217.

Caffrey, M. and Cherezov, V. (2009). Crystallizing membrane proteins using lipidic mesophases. Nature protocols *4*, 706–31.

Chen, V.B., Arendall, W.B., Headd, J.J., Keedy, D.a., Immormino, R.M., Kapral, G.J., Murray, L.W., Richardson, J.S., and Richardson, D.C. (2010). MolProbity: all-atom structure validation for macromolecular crystallography. Acta Cryst D *66*, 12–21.

Cherezov, V., Peddi, A., Muthusubramaniam, L., Zheng, Y.F., and Caffrey, M. (2004). A robotic system for crystallizing membrane and soluble proteins in lipidic mesophases. Acta Cryst D *60*, 1795–807.

Collaborative Computational Project, N.. (1994). The CCP4 suite: programs for protein crystallography. Acta Cryst D *50*, 760–3.

Dahl, A.C.E., Chavent, M., and Sansom, M.S.P. (2012). Bendix: intuitive helix geometry analysis and abstraction. Bioinformatics *28*, 2193–4.

Dang, S., Sun, L., Huang, Y., Lu, F., Liu, Y., Gong, H., Wang, J., and Yan, N. (2010). Structure of a fucose transporter in an outward-open conformation. Nature *467*, 734–8.

Deng, D., Xu, C., Sun, P., Wu, J., Yan, C., Hu, M., and Yan, N. (2014). Crystal structure of the human glucose transporter GLUT1. Nature *510*, 121–5.

Doki, S., Kato, H.E., Solcan, N., Iwaki, M., Koyama, M., Hattori, M., Iwase, N., Tsukazaki, T., Sugita, Y., Kandori, H., et al. (2013). Structural basis for dynamic mechanism of proton-coupled symport by the peptide transporter POT. Proc Natl Acad Sci U S A *110*, 11343–8.

Drew, D., Lerch, M., Kunji, E., Slotboom, D.J., and de Gier, J.W. (2006). Optimization of membrane protein overexpression and purification using GFP fusions. Nature Methods *3*, 303–13.

Emsley, P., Lohkamp, B., Scott, W.G., and Cowtan, K. (2010). Features and development of Coot. Acta Cryst D *66*, 486–501.

Evans, P.R. (2011). An introduction to data reduction: space-group determination, scaling and intensity statistics. Acta Cryst D *67*, 282–92.

Frishman, D. and Argos, P. (1995). Knowledge-based protein secondary structure assignment. Proteins *23*, 566–79.

Guettou, F., Quistgaard, E.M., Trésaugues, L., Moberg, P., Jegerschöld, C., Zhu, L., Jong, A.J.O., Nordlund, P., and Löw, C. (2013). Structural insights into substrate recognition in proton-dependent oligopeptide transporters. EMBO reports *14*, 804–10.

Huang, Y., Lemieux, M.J., Song, J., Auer, M., and Wang, D.N. (2003). Structure and mechanism of the glycerol-3-phosphate transporter from Escherichia coli. Science *301*, 616–20.

Humphrey, W., Dalke, A., and Schulten, K. (1996). VMD: visual molecular dynamics. J Mol Graph *14*, 33–38.

Jeschke, G., Chechik, V., Ionita, P., Godt, A., Zimmermann, H., Banham, J., Timmel, C.R., Hilger, D., and Jung, H. (2006). DeerAnalysis2006 - a comprehensive software package for analyzing pulsed ELDOR data. Appl Mag Reson *30*, 473–498.

Jiang, D., Zhao, Y., Wang, X., Fan, J., Heng, J., Liu, X., Feng, W., Kang, X., Huang, B., Liu, J., et al. (2013). Structure of the YajR transporter suggests a transport mechanism based on the conserved motif A. Proc Natl Acad Sci U S A *110*, 14664–9.

Kabsch, W. (2010). XDS. Acta Cryst D *66*, 125–32.

Laskowski, R.A., MacArthur, M.W., Moss, D.S., and Thornton, J.M. (1993). PROCHECK: a program to check the stereochemical quality of protein structures. J Appl Cryst *26*, 283–291.

Li, D., Boland, C., Aragao, D., Walsh, K., and Caffrey, M. (2012). Harvesting and cryo-cooling crystals of membrane proteins grown in lipidic mesophases for structure determination by macromolecular crystallography. JoVE e4001.

Lovett, J.E., Lovett, B.W., and Harmer, J. (2012). DEER-Stitch: combining three- and four-pulse DEER measurements for high sensitivity, deadtime free data. J Mag Res *223*, 98–106.

Lyons, J.A., Parker, J.L., Solcan, N., Brinth, A., Li, D., Shah, S.T., Caffrey, M., and Newstead, S. (2014). Structural basis for polyspecificity in the POT family of proton-coupled oligopeptide transporters. EMBO reports *15*, 886–93.

MacKerell, A.D., Bashford, D., Dunbrack, R.L., Evanseck, J.D., Field, M.J., Fischer, S., Gao, J., Guo, H., Ha, S., Joseph-McCarthy, D., et al. (1998). All-Atom Empirical Potential for Molecular Modeling and Dynamics Studies of Proteins. J Phys Chem B *102*, 3586–3616.

Martínez, L., Andreani, R., and Martínez, J.M. (2007). Convergent algorithms for protein structural alignment. BMC bioinformatics *8*, 306.

McCoy, A.J., Grosse-Kunstleve, R.W., Adams, P.D., Winn, M.D., Storoni, L.C., and Read, R.J. (2007). Phaser crystallographic software. J Appl Cryst *40*, 658–674.

Michaud-Agrawal, N., Denning, E.J., Woolf, T.B., and Beckstein, O. (2011). MDAnalysis: A toolkit for the analysis of molecular dynamics simulations. J Comput Chem *32*, 2319–2327.

Milov, A.D., Salikhov, K.M., and Shirov, M.D. (1981). Application of ELDOR in electron- spin echo for paramagnetic center space distribution in solids. Fiz Tverd Tela *23*, 975–982.

Newstead, S., Drew, D., Cameron, A.D., Postis, V.L.G., Xia, X., Fowler, P.W., Ingram, J.C., Carpenter, E.P., Sansom, M.S.P., McPherson, M.J., et al. (2011). Crystal structure of a prokaryotic homologue of the mammalian oligopeptide-proton symporters, PepT1 and PepT2. EMBO J *30*, 417–426.

Pannier, M., Veit, S., Godt, A., Jeschke, G., and Spiess, H.W. (2000). Dead-time free measurement of dipole-dipole interactions between electron spins. J Mag Res *142*, 331–40.

Parker, J.L. and Newstead, S. (2014). Molecular basis of nitrate uptake by the plant nitrate transporter NRT1.1. Nature *507*, 68–72.

Pedersen, B.P., Kumar, H., Waight, A.B., Risenmay, A.J., Roe-Zurz, Z., Chau, B.H., Schlessinger, A., Bonomi, M., Harries, W., Sali, A., et al. (2013). Crystal structure of a eukaryotic phosphate transporter. Nature *496*, 533–6.

Petrey, D., Xiang, Z., Tang, C.L., Xie, L., Gimpelev, M., Mitros, T., Soto, C.S., Goldsmith-Fischman, S., Kernytsky, A., Schlessinger, A., et al. (2003). Using multiple structure alignments, fast model building, and energetic analysis in fold recognition and homology modeling. Proteins *53 Suppl 6*, 430–5.

Polyhach, Y., Bordignon, E., and Jeschke, G. (2011). Rotamer libraries of spin labelled cysteines for protein studies. Phys Chem Chem Phys *13*, 2356–66.

Pronk, S., Páll, S., Schulz, R., Larsson, P., Bjelkmar, P., Apostolov, R., Shirts, M.R., Smith, J.C., Kasson, P.M., van der Spoel, D., et al. (2013). GROMACS 4.5: a high-throughput and highly parallel open source molecular simulation toolkit. Bioinformatics *29*, 845–54.

Quistgaard, E.M., Löw, C., Moberg, P., Trésaugues, L., and Nordlund, P. (2013). Structural basis for substrate transport in the GLUT-homology family of monosaccharide transporters. Nat Struct Mol Biol *20*, 766–8.

Sali, A. and Blundell, T.L. (1993). Comparative protein modelling by satisfaction of spatial restraints. J Mol Biol *234*, 779–815.

Smart, O.S., Neduvelil, J.G., Wang, X., Wallace, B.A., and Sansom, M.S.P. (1996). HOLE: a program for the analysis of the pore dimensions of ion channel structural models. J Mol Graph *14*, 354–60.

Solcan, N., Kwok, J., Fowler, P.W., Cameron, A.D., Drew, D., Iwata, S., and Newstead, S. (2012). Alternating access mechanism in the POT family of oligopeptide transporters. EMBO J *31*, 3411–21.

Stelzl, L.S., Fowler, P.W., Sansom, M.S., and Beckstein, O. (2014). Flexible Gates Generate Occluded Intermediates in the Transport Cycle of LacY. J Mol Biol *426*, 735–751.

Sun, L., Zeng, X., Yan, C., Sun, X., Gong, X., Rao, Y., and Yan, N. (2012). Crystal structure of a bacterial homologue of glucose transporters GLUT14. Nature *490*, 361–366.

Winter, G., Lobley, C.M.C., and Prince, S.M. (2013). Decision making in xia2. Acta Cryst D *69*, 1260–73.

Word, J.M., Lovell, S.C., Richardson, J.S., and Richardson, D.C. (1999). Asparagine and glutamine: using hydrogen atom contacts in the choice of side-chain amide orientation. J Mol Biol *285*, 1735–47.

Yan, H., Huang, W., Yan, C., Gong, X., Jiang, S., Zhao, Y., Wang, J., and Shi, Y. (2013). Structure and Mechanism of a Nitrate Transporter. Cell Rep *3*, 716–723.

Yin, Y., He, X., Szewczyk, P., Nguyen, T., and Chang, G. (2006). Structure of the multidrug transporter EmrD from Escherichia coli. Science *312*, 741–4.
